# Treatment shortening and relapse control in drug-susceptible pulmonary tuberculosis: a network meta-analysis of randomized trials

**DOI:** 10.3389/fcimb.2026.1869454

**Published:** 2026-09-03

**Authors:** Fengjie Tang, Juan Li, Qihuan Ma, Shasha Tao, Ling Zhou, Yanmei Feng

**Affiliations:** 1 Department of Respiratory Medicine, Chongqing Emergency Medical Center, Chongqing University Central Hospital, Chongqing Key Laboratory of Emergency Medicine, Chongqing, China; 2 Department of Pulmonary and Critical Care Medicine, Chongqing Liang Jiang New Area Traditional Chinese Medicine Hospital, Chongqing, China; 3 Chongqing Key Laboratory of Emergency Medicine, Chongqing Emergency Medical Center, Chongqing University Central Hospital, Chongqing, Chongqing, China; 4 Department of Thoracic Surgery, Chongqing Public Health Medical Center, Chongqing, China

**Keywords:** drug-susceptible pulmonary tuberculosis, *Mycobacterium tuberculosis*, network meta-analysis, regimen architecture, treatment shortening

## Abstract

Drug-susceptible pulmonary tuberculosis (DS-PTB) is conventionally treated with a 6-month multidrug regimen, but prolonged treatment may compromise adherence, prompting efforts to develop shorter and effective therapeutic strategies. However, the comparative long-term effectiveness of alternative regimens, particularly their ability to prevent relapse, remains uncertain. We systematically searched multiple databases from inception through August 31, 2025, for randomized controlled trials enrolling adolescents and adults with DS-PTB. Regimens were classified according to treatment duration and architecture, including standard 6-month therapy, rifamycin-intensified strategies, structurally modified 6-month regimens, 4-month treatment-shortening approaches, and intended 8-week multidrug regimens. A random-effects pairwise meta-analysis and a fixed-effect consistency network meta-analysis (NMA) compared treatment success, relapse/recurrence-related outcomes, and mortality. Eight randomized controlled trials involving 10,244 participants were included. Six source trials involving 8,457 randomized participants contributed to the quantitative synthesis and informed a 14-node NMA; outcome data from 6,752 participants contributed to 12 shortened-regimen-versus-standard comparisons in the pairwise meta-analysis. Two trials involving 1,787 participants were summarized narratively because their thrice-weekly control regimens were not considered exchangeable with the daily reference regimen. In the pooled pairwise analysis, shortened-duration regimens were associated with a higher risk of trial-defined unfavorable long-term outcomes than the 6-month standard regimen (RR = 1.68, 95% CI 1.37–2.06; P < 0.001; I² = 39.9%). Treatment success was close to that of the standard regimen for the 4-month daily rifapentine-moxifloxacin regimen evaluated in Study 31/A5349 (RR = 0.98, 95% CI 0.94–1.01) and the 6-month regimen with once-weekly rifapentine-moxifloxacin continuation therapy evaluated in RIFAQUIN (RR = 1.01, 95% CI 0.93–1.09). In contrast, all four intended 8-week TRUNCATE-TB regimens showed lower treatment success and higher relapse/recurrence-related risk estimates than the standard regimen. Mortality was uncommon, and mortality comparisons were imprecise. These findings suggest that long-term outcomes should be interpreted in relation to regimen design, dosing, and treatment duration rather than duration alone. The standard 6-month regimen remains a reliable reference, and conclusions about individual alternatives should be based on effect estimates and uncertainty rather than rankings. Because no closed loop was formed by independent trial evidence, formal inconsistency assessment was not feasible.

**Systematic review registration:**

https://www.crd.york.ac.uk/PROSPERO/, identifier CRD420251267175.

## 1 Introduction

Tuberculosis remains a major global public health burden, with drug-susceptible pulmonary tuberculosis (DS-PTB) accounting for the vast majority of newly diagnosed cases worldwide (World Health Organization, 2025). The 6-month regimen remains an established reference regimen, while the 4-month rifapentine-moxifloxacin regimen is recommended for eligible patients under specified conditions (Saukkonen et al., 2025; World Health Organization, 2025). The durability of tuberculosis treatment is thought to depend partly on sustained sterilizing activity against persistent *Mycobacterium tuberculosis* subpopulations within pulmonary lesions, providing a biological rationale for concern about relapse when treatment is shortened (Barry et al., 2009; Mitchison and Davies, 2012).

To improve treatment adherence and outcomes, alternative strategies—including treatment-shortening strategies and phase-specific regimen modifications—have been evaluated in randomized controlled trials (Chang et al., 2024; Dorman et al., 2021). However, their relative efficacy and long-term outcomes remain inconsistent, and systematic comparisons across competing regimens are lacking (Chang et al., 2024; Dorman et al., 2021).

Longer treatment duration may increase treatment burden and adversely affect adherence in some settings (Singh, 2024). Accordingly, multiple treatment strategies have been explored to simplify therapy while maintaining efficacy (Imperial et al., 2018; Nahid et al., 2016; Saukkonen et al., 2025). Although some approaches yield favorable early bacteriological responses (Dorman et al., 2021), long-term outcomes—particularly relapse control—remain inconsistent across studies (Grace et al., 2019; Imperial et al., 2018). Moreover, prior reviews have not fully resolved the comparative long-term performance of increasingly diverse treatment-shortening strategies, partly because intervention designs and outcome definitions vary across trials (Grace et al., 2019; Imperial et al., 2018; Menzies et al., 2009).

Network meta-analysis (NMA) enables integrated comparisons of multiple competing treatment strategies by synthesizing both direct and indirect evidence (Brignardello-Petersen and Guyatt, 2025; Florez et al., 2024). We therefore conducted a NMA of randomized trials to compare long-term outcomes across DS-PTB treatment strategies differing in treatment duration and regimen design, with microbiological factors considered in the interpretation of relapse control.

## 2 Methods

This study was an NMA comparing the relative effectiveness of anti-tuberculosis treatment strategies in patients with DS-PTB. A prespecified analytical framework was applied, with the study population, interventions, outcomes, and statistical methods defined *a priori* to minimize data-driven bias. The protocol was registered in the International Prospective Register of Systematic Reviews (PROSPERO; CRD420251267175), and the study was conducted and reported in accordance with the Preferred Reporting Items for Systematic Reviews and Meta-Analyses (PRISMA) 2020 guidelines (Page et al., 2021). The wording of the manuscript title was refined after study completion to better reflect the scope of the analysis, without changes to the registered protocol.

### 2.1 Search strategy and information sources

A systematic literature search was conducted from database inception to August 31, 2025, in MEDLINE (via PubMed), Embase, the Cochrane Central Register of Controlled Trials (CENTRAL), Web of Science Core Collection, Scopus, the China National Knowledge Infrastructure (CNKI), and Wanfang Data. ClinicalTrials.gov, the World Health Organization (WHO) International Clinical Trials Registry Platform (ICTRP), reference lists of included studies and relevant systematic reviews, and grey literature were also searched. Search results from Scopus, Wanfang Data, ClinicalTrials.gov, and WHO ICTRP were cross-checked against the master database but yielded no additional unique records for screening after cross-source deduplication. The search strategy followed the Population, Intervention, Comparator, Outcomes, and Study design framework. Validated randomized controlled trial filters were applied in PubMed and Embase; no methodological filter was required for CENTRAL or applied to the other databases. No language or publication date restrictions were imposed. Further details of the search strategy, record management, and study-selection procedures are provided in **Methods S1**–**S3**.

### 2.2 Study selection and eligibility criteria

All retrieved records were managed using combined automated and manual deduplication procedures before screening. Records supporting bulk citation export were imported into a single EndNote library, whereas records from sources without compatible export functions were entered into a structured screening spreadsheet and cross-checked against the EndNote library. Duplicates and near-duplicates were identified automatically and verified manually, and multiple reports of the same trial were linked to a single study record. Full texts were retrieved for records considered potentially eligible after title and abstract screening. Two reviewers (FT and JL) independently screened titles and abstracts and subsequently assessed full-text reports. Disagreements were resolved through discussion or adjudication by a third reviewer (QM), and reasons for full-text exclusion were documented. Further details are provided in **Methods S3**.

Eligible studies were randomized controlled trials (RCTs) enrolling adults or adolescents with microbiologically confirmed DS-PTB. Eligible interventions were predefined treatment-shortening strategies, including fixed regimens of ≤4 months, modified 6-month regimens with altered continuation phases, and strategy-based regimens with response-guided total duration. Comparators were standard 6-month regimens consisting of isoniazid, rifampicin, pyrazinamide, and ethambutol (HRZE) followed by isoniazid and rifampicin (HR), or equivalent regimens. Studies were required to report at least one relevant outcome, including treatment success or failure, relapse or recurrence with ≥12 months of follow-up, all-cause mortality, or grade ≥3 adverse events. Studies were excluded if they focused on multidrug-resistant or rifampicin-resistant tuberculosis; enrolled exclusively extrapulmonary tuberculosis or pediatric populations; were nonrandomized; did not evaluate treatment-shortening strategies or predefined regimen modifications; or had insufficient follow-up or unavailable outcome data. Detailed eligibility criteria are summarized in **Table 1**.

**Table 1:** Eligibility criteria according to the PICOS framework.

Domain	Inclusion criteria	Exclusion criteria
Population	Adults or adolescents with microbiologically confirmed drug-susceptible pulmonary tuberculosis.	Multidrug-resistant or rifampicin-resistant tuberculosis; exclusively extrapulmonary tuberculosis; pediatric-only cohorts.
Intervention	Treatment-shortening or strategy-modified regimens, including fixed regimens of ≤4 months; 6-month regimens with substantively modified continuation phases; and response-guided strategies with an 8-week initial intensive phase (e.g., TRUNCATE-TB).	Regimens not intended to shorten treatment; dosing or pharmacokinetic studies without a predefined shortened duration.
Comparator	Standard 6-month regimen: HRZE followed by HR, or an equivalent regimen.	No active concurrent comparator; historical controls only.
Outcomes	Treatment success or failure; relapse or recurrence assessed ≥12 months after randomization or treatment completion; all-cause mortality; or grade ≥3 adverse events.	No prespecified clinical outcome data; <12 months of follow-up for relapse or recurrence.
Design	Randomized controlled trials, irrespective of masking.	Nonrandomized, single-arm, or observational studies.

PICOS, Population, Intervention, Comparator, Outcomes, and Study design; HRZE, isoniazid, rifampicin, pyrazinamide, and ethambutol; HR, isoniazid and rifampicin.

### 2.3 Data extraction and risk of bias assessment

Two reviewers (FT and JL) independently extracted study characteristics, participant information, intervention and comparator details, follow-up duration, and outcome definitions using a standardized form. Because the trials used different estimands and analysis populations, the NMA used the trial-reported primary or assessable efficacy population for treatment success and relapse/recurrence-related outcomes and the broadest reported randomized or safety population for mortality; outcome-specific definitions and denominators are reported in **Supplementary Table 3**. For multi-arm trials, all eligible randomized arms were retained; shared comparator groups were divided where required in pairwise meta-analyses, whereas correlations induced by shared comparators were retained within the network model to avoid double-counting and preserve the appropriate correlation structure. Risk of bias was independently assessed by the same reviewers using the Cochrane Risk of Bias tool (RoB 2) across five domains: the randomization process, deviations from intended interventions, missing outcome data, outcome measurement, and selection of the reported result (Sterne et al., 2019). RoB 2 judgements were outcome-specific; **Supplementary Figure 6** summarizes the primary long-term efficacy result used from each trial. Disagreements were resolved through discussion or adjudication by a third reviewer (QM) when necessary.

### 2.4 Treatment node construction and assessment of transitivity

Given heterogeneity in treatment duration, drug composition, rifamycin dose, dosing frequency, continuation-phase structure, and implementation across trials, interventions were classified into clinically defined regimen-level nodes (Cipriani et al., 2013; Salanti, 2012). Regimens were retained as separate nodes when clinically relevant differences in composition, dose, or administration schedule could affect efficacy or relapse risk. Fourteen treatment nodes were used in the NMA: the 6-month standard regimen; the 4-month daily rifapentine-moxifloxacin regimen and 4-month daily rifapentine regimen evaluated in Study 31/A5349; the two 4-month moxifloxacin-substitution regimens evaluated in REMoxTB; the 4-month gatifloxacin-containing regimen evaluated in OFLOTUB; the 4-month rifapentine-moxifloxacin regimen with twice-weekly continuation and the 6-month regimen with once-weekly continuation evaluated in RIFAQUIN; the 4-month 1,200-mg and 1,800-mg daily rifampicin regimens evaluated in RIFASHORT; and four intended 8-week TRUNCATE-TB regimens comprising high-dose rifampicin-linezolid, high-dose rifampicin-clofazimine, rifapentine-linezolid-levofloxacin, and bedaquiline-linezolid combinations. Protocol-permitted extension beyond 8 weeks in TRUNCATE-TB was regarded as part of the regimen implementation and was considered in the indirectness assessment.

A trial arm was mapped when the trial included the daily 6-month standard regimen defining the reference node and the experimental regimen met one of the clinically defined regimen-level node definitions. The prespecified 2025 TRUNCATE-TB regimen-efficacy analysis reported conventional participant-level outcomes separately for each intended 8-week regimen and the 24-week standard arm; these data were therefore mapped to four regimen-specific nodes. This estimand is distinct from the response-guided management-strategy estimand reported previously, which incorporated surveillance and protocol-defined re-treatment (Paton et al., 2023, Paton et al., 2025). Jawahar et al. (2013) and Velayutham et al. (2020) used thrice-weekly 6-month controls, which were not considered exchangeable with daily HRZE followed by HR because intermittency alters rifamycin exposure and potentially relapse risk; their arms were retained in the systematic review and narrative synthesis but were not mapped to the quantitative network. Transitivity was assessed by comparing trial eligibility criteria, baseline disease severity, human immunodeficiency virus (HIV) status, follow-up duration, outcome definitions, dosing frequency, and regimen implementation. Complete arm-to-node mapping and reasons for non-mapping are reported in **Supplementary Table 2**.

### 2.5 Statistical analysis

All analyses were performed using R version 4.3.3. Pairwise meta-analyses were performed on the log-risk-ratio scale using inverse-variance random-effects models, with between-comparison heterogeneity estimated using the DerSimonian–Laird method (DerSimonian and Laird, 1986). Relative effects were reported as risk ratios (RRs) with 95% confidence intervals (CIs). The primary pairwise synthesis included shortened-duration regimens versus the daily 6-month standard regimen. Shared control groups in multi-arm trials were divided equally across eligible comparisons for weighting while retaining the original control risk; original control counts are displayed in the forest plot and marked accordingly. Heterogeneity was summarized using I² and Cochran’s Q test (Higgins and Thompson, 2002). Sensitivity analyses used a fixed-effect model, the Hartung–Knapp–Sidik–Jonkman method (IntHout et al., 2014), and exclusion of TRUNCATE-TB to examine the influence of the intended 8-week regimens. The NMA was fitted as a frequentist fixed-effect consistency model on the log-risk-ratio scale. Multi-arm covariance was retained to account for correlations induced by shared comparators. No continuity correction was used for treatment success or relapse/recurrence-related outcomes; because two TRUNCATE-TB arms reported zero deaths, a 0.5 correction was applied to every TRUNCATE-TB arm and its common standard comparator in the mortality model.

Global and local inconsistency assessments were prespecified using the design-by-treatment interaction test and node-splitting, respectively (Higgins et al., 2012; Dias et al., 2010). However, all independent evidence was anchored on the standard regimen, and the structural triangles generated by the five multi-arm trials were consistent by construction rather than independent closed loops. Consequently, the design-by-treatment test had zero degrees of freedom and node-splitting was not estimable. The 14-node network provided 13 independent basic contrasts, exactly one fewer than the number of nodes, leaving no residual degrees of freedom for estimating a common network heterogeneity variance; the fixed-effect model was therefore retained. P-scores were calculated descriptively and interpreted only alongside relative-effect estimates and confidence intervals (Rücker and Schwarzer, 2015). Publication bias was explored visually, but formal asymmetry tests were not conducted because fewer than 10 independent source trials were available.

### 2.6 Certainty of evidence assessment

The certainty of evidence for the primary outcomes was assessed using an adapted approach based on the Confidence in Network Meta-Analysis (CINeMA) framework (Nikolakopoulou et al., 2020), hereafter termed a CINeMA-style assessment. Two reviewers independently applied the CINeMA domains, with disagreements resolved through discussion. Because the CINeMA web application was not used and no contribution matrix was calculated, “CINeMA-style” is used consistently throughout the main text, figure legends, and **Supplementary Material** to distinguish this approach from a formal CINeMA assessment. The framework was selected because it extends the Grading of Recommendations Assessment, Development and Evaluation (GRADE) approach to NMA by incorporating network-specific domains, including indirectness and incoherence. The adapted assessment considered within-study bias, reporting bias, indirectness, imprecision, and heterogeneity. Because the network contained no closed loop formed by independent trials, the incoherence domain could not be rated and was recorded as not applicable rather than “not serious” to avoid inflating the overall certainty. Overall certainty was rated as high, moderate, low, or very low using conventional GRADE categories.

## 3 Results

### 3.1 Study selection and study characteristics

**Figure 1:**
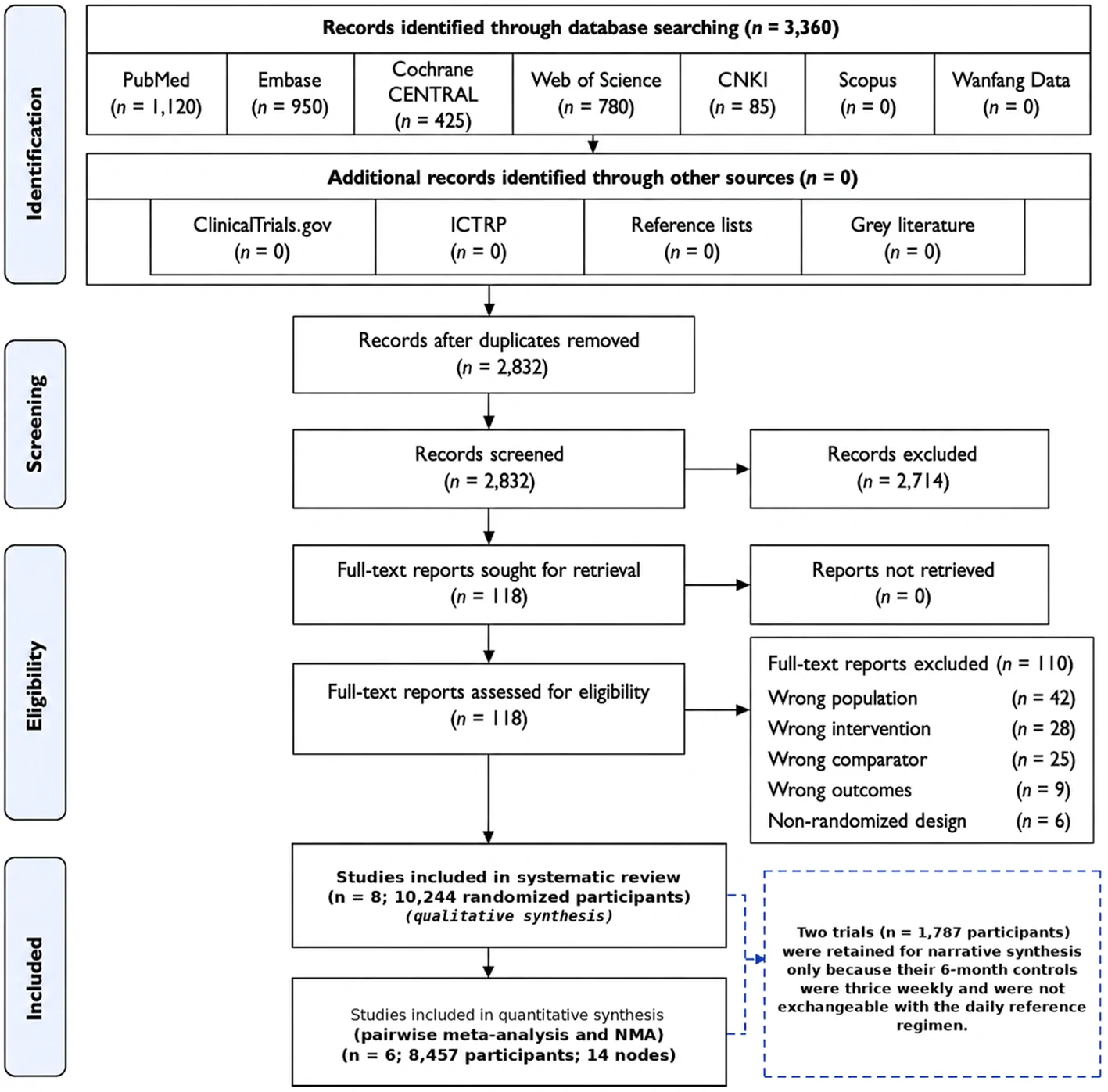
PRISMA flow diagram. Eight randomized controlled trials (10,244 randomized participants) were included; six trials (8,457 participants) contributed to pairwise and network meta-analysis, and two trials (1,787 participants) were retained for narrative synthesis because their standard controls were thrice weekly. Abbreviations: CNKI, China National Knowledge Infrastructure; ICTRP, International Clinical Trials Registry Platform; NMA, network meta-analysis; n, number.

The systematic search identified 3,360 records: PubMed (n = 1,120), Embase (n = 950), CENTRAL (n = 425), Web of Science (n = 780), and CNKI (n = 85). Search results from Scopus, Wanfang Data, ClinicalTrials.gov, and WHO ICTRP were cross-checked against the master database but yielded no additional unique records for screening after cross-source deduplication. After removal of 528 duplicates, 2,832 records underwent title and abstract screening, and 118 full-text reports were assessed for eligibility. Of these, 110 were excluded for the reasons shown in **Figure 1**. Eight RCTs met the eligibility criteria: Dorman et al. (2021); Gillespie et al. (2014); Jawahar et al. (2013); Jindani et al. (2014); Jindani et al. (2023); Merle et al. (2014); Paton et al. (2025), and Velayutham et al. (2020).

The eight RCTs included 10,244 randomized participants across Africa, Asia, and Latin America. Six source trials (Dorman et al., 2021; Gillespie et al., 2014; Jindani et al., 2014, Jindani et al., 2023; Merle et al., 2014; Paton et al., 2025), involving 8,457 randomized participants, contributed to the quantitative synthesis. Their outcome-specific data comprised 6,752 participants in the pooled pairwise analysis. Paton et al. (2025) contributed four intended 8-week regimen comparisons because the prespecified regimen-efficacy report supplied conventional arm-level outcomes separately from the broader management-strategy estimand. Jawahar et al. (2013) and Velayutham et al. (2020), involving 1,787 participants, were retained for narrative synthesis only because their 6-month controls were administered thrice weekly rather than daily. Study characteristics and quantitative contributions are summarized in **Table 2**, with complete arm mapping in **Supplementary Table 2**.

**Table 2:** Characteristics of the included randomized trials and their contribution to the quantitative synthesis.

Study	Region(s) and setting	Intervention and comparator	Randomized, n	Follow-up and primary outcome	Key findings relevant to this review	Contribution to the quantitative synthesis
Dorman et al., 2021 (Study 31/A5349)	13 countries; multicenter	Two 4-month rifapentine-containing regimens (with or without moxifloxacin) versus the standard 6-month regimen.	2,516	Tuberculosis disease-free survival at 12 months after randomization.	The 4-month rifapentine–moxifloxacin regimen was non-inferior to the standard regimen; the rifapentine-only regimen was not.	Included; two arms mapped (4-month RPT+MOX; 4-month RPT)
Gillespie et al., 2014 (REMoxTB)	9 countries; double-blind	Two 4-month moxifloxacin-substitution regimens versus the standard 6-month regimen.	1,931	Treatment failure or relapse within 18 months after randomization.	Neither 4-month moxifloxacin regimen was non-inferior to the standard regimen.	Included; two arms mapped (4-month MOX, H-subst; 4-month MOX, E-subst)
Merle et al., 2014 (OFLOTUB)	Five sub-Saharan African countries	4-month gatifloxacin-containing regimen versus the standard 6-month regimen.	1,836	Unfavorable outcome at 24 months after treatment completion.	The 4-month gatifloxacin regimen was inferior to the standard regimen.	Included; one arm mapped (4-month GAT)
Jindani et al., 2014 (RIFAQUIN)	Southern Africa	4-month and 6-month rifapentine–moxifloxacin regimens versus the standard 6-month regimen.	827	Composite treatment failure or relapse; modified intention-to-treat and per-protocol analyses.	The 6-month once-weekly rifapentine–moxifloxacin continuation regimen was non-inferior; the 4-month regimen was not.	Included; two arms mapped (4-month RPT+MOX, daily; 6-month RPT+MOX, weekly continuation)
Velayutham et al., 2020	India; multicenter	Four 3- or 4-month moxifloxacin-containing regimens versus a standard 6-month thrice-weekly control regimen.	1,371 (mITT, 1,329; PP, 1,223)	Tuberculosis recurrence during 24 months after treatment completion; end-of-treatment response.	The daily 4-month moxifloxacin regimen demonstrated equivalence to the control; other 4-month regimens did not.	Not included; the thrice-weekly control regimen is not exchangeable with the daily reference node
Jawahar et al., 2013	India (Chennai and Madurai)	4-month thrice-weekly moxifloxacin or gatifloxacin regimens versus a 6-month thrice-weekly control regimen.	416	Tuberculosis recurrence during 24 months after treatment completion; end-of-treatment response.	The Data and Safety Monitoring Board recommended early termination because of high recurrence in the 4-month arms.	Not included; the thrice-weekly control regimen is not exchangeable with the daily reference node
Jindani et al., 2023 (RIFASHORT)	Africa, South Asia, and Peru	Two 4-month high-dose rifampicin regimens (1,200 mg or 1,800 mg) versus the standard 6-month regimen.	672	Composite unfavorable outcome (treatment failure or relapse) with ≥12 months of follow-up.	Neither 4-month high-dose rifampicin regimen was non-inferior to the standard regimen.	Included; two arms mapped (4-month RIF1200; 4-month RIF1800)
Paton et al., 2025 (TRUNCATE-TB)	Asia and Africa; multicenter	Four intended 8-week multidrug regimens versus the standard 24-week regimen; protocol-permitted extension was response guided.	675 (674 in ITT)	Prespecified regimen-efficacy analysis of unfavorable outcome, treatment success, relapse, and safety.	All intended 8-week regimens had lower conventional efficacy than the standard, mainly because of relapse; this estimand differs from management-strategy effectiveness with surveillance and re-treatment.	Included; four regimen-specific nodes (TR-RIFLZD, TR-RIFCLO, TR-RPTLZDLVX, TR-BDQLZD) plus standard reference

BDQ, bedaquiline; CI, confidence interval; CLO, clofazimine; GAT, gatifloxacin; ITT, intention-to-treat; LVX, levofloxacin; LZD, linezolid; mITT, modified intention-to-treat; MOX, moxifloxacin; NMA, network meta-analysis; PP, per-protocol; RIF, rifampicin; RPT, rifapentine. Six source trials (8,457 randomized participants) informed the 14-node NMA; outcome data from 6,752 participants (4,298 shortened-regimen and 2,454 comparator participants) contributed to 12 pooled shortened-regimen comparisons. Two trials (1,787 participants) were retained for narrative synthesis only. Complete arm-to-node mapping is provided in **Supplementary Table 2**.

### 3.2 Pairwise meta-analysis

**Figure 2:**
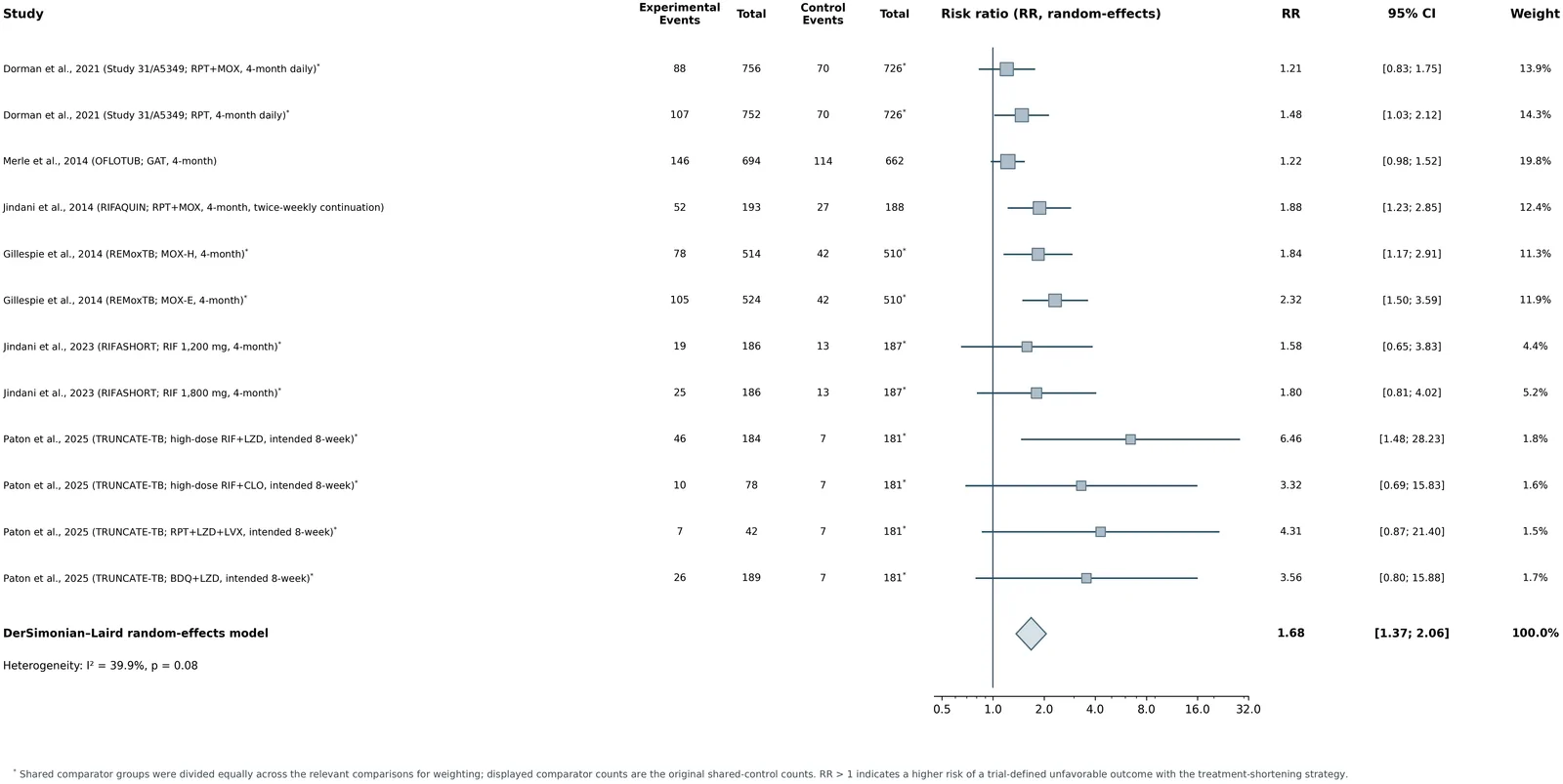
Forest plot of 12 shortened-regimen-versus-standard comparisons of trial-defined unfavorable long-term outcomes using the DerSimonian-Laird random-effects model. Six source trials randomized 8,457 participants; outcome data from 6,752 participants (4,298 shortened-regimen and 2,454 comparator participants) informed the pooled estimate. Shared controls from multi-arm trials were divided equally for weighting while original event counts are displayed and marked with a superscript asterisk. The modified 6-month RIFAQUIN arm is not part of this shortened-duration pool but is included in the NMA. RR > 1 favors the 6-month standard regimen. Abbreviations: BDQ, bedaquiline; CI, confidence interval; CLO, clofazimine; DL, DerSimonian–Laird; LVX, levofloxacin; LZD, linezolid; MOX, moxifloxacin; RIF, rifampicin; RPT, rifapentine; RR, risk ratio.

Six of the eight included RCTs randomized 8,457 participants and contributed 12 shortened-regimen-versus-standard comparisons to the pairwise meta-analysis: two each from Study 31/A5349, REMoxTB, and RIFASHORT; one each from OFLOTUB and RIFAQUIN; and four from TRUNCATE-TB. Outcome data were available for 6,752 participants (4,298 shortened-regimen participants and 2,454 comparator participants). The random-effects model showed a higher risk of trial-defined unfavorable long-term outcomes with shortened regimens than with the 6-month standard regimen (RR = 1.68, 95% CI 1.37–2.06; P < 0.001; **Figure 2**). Heterogeneity was moderate (I² = 39.9%; Q-test P = 0.08). The fixed-effect estimate was RR = 1.52 (95% CI 1.33–1.74). Shared standard groups from multi-arm trials were divided equally for weighting, without altering the original event risks.

Strategy-specific estimates varied substantially. Existing 4-month regimens ranged from the Study 31/A5349 daily rifapentine-moxifloxacin regimen, whose estimate was closest to the standard, to regimens with clearly higher unfavorable-outcome risks. All four intended 8-week TRUNCATE-TB regimens favored the standard regimen: high-dose rifampicin-linezolid RR = 6.46 (95% CI 1.48–28.23), high-dose rifampicin-clofazimine RR = 3.32 (95% CI 0.69–15.83), rifapentine-linezolid-levofloxacin RR = 4.31 (95% CI 0.87–21.40), and bedaquiline-linezolid RR = 3.56 (95% CI 0.80–15.88). Their wide pairwise confidence intervals reflect the small common standard arm after multi-arm weighting, but the directions were concordant with the regimen-level outcome data and NMA estimates (**Figure 2**; **Supplementary Table 3**). The 6-month once-weekly RIFAQUIN regimen was not included in this shortened-duration pool but remained a node in the NMA.

Sensitivity analyses were consistent with the primary result (**Supplementary Figure 2**; **Supplementary Table 1**). The fixed-effect estimate was RR = 1.52 (95% CI 1.33–1.74), and the Hartung–Knapp–Sidik–Jonkman method produced RR = 1.68 (95% CI 1.34–2.10). Excluding TRUNCATE-TB yielded RR = 1.54 (95% CI 1.29–1.85) for the five conventional 4-month trials; the direction of the pooled association was therefore unchanged.

### 3.3 Evidence network structure

**Figure 3:**
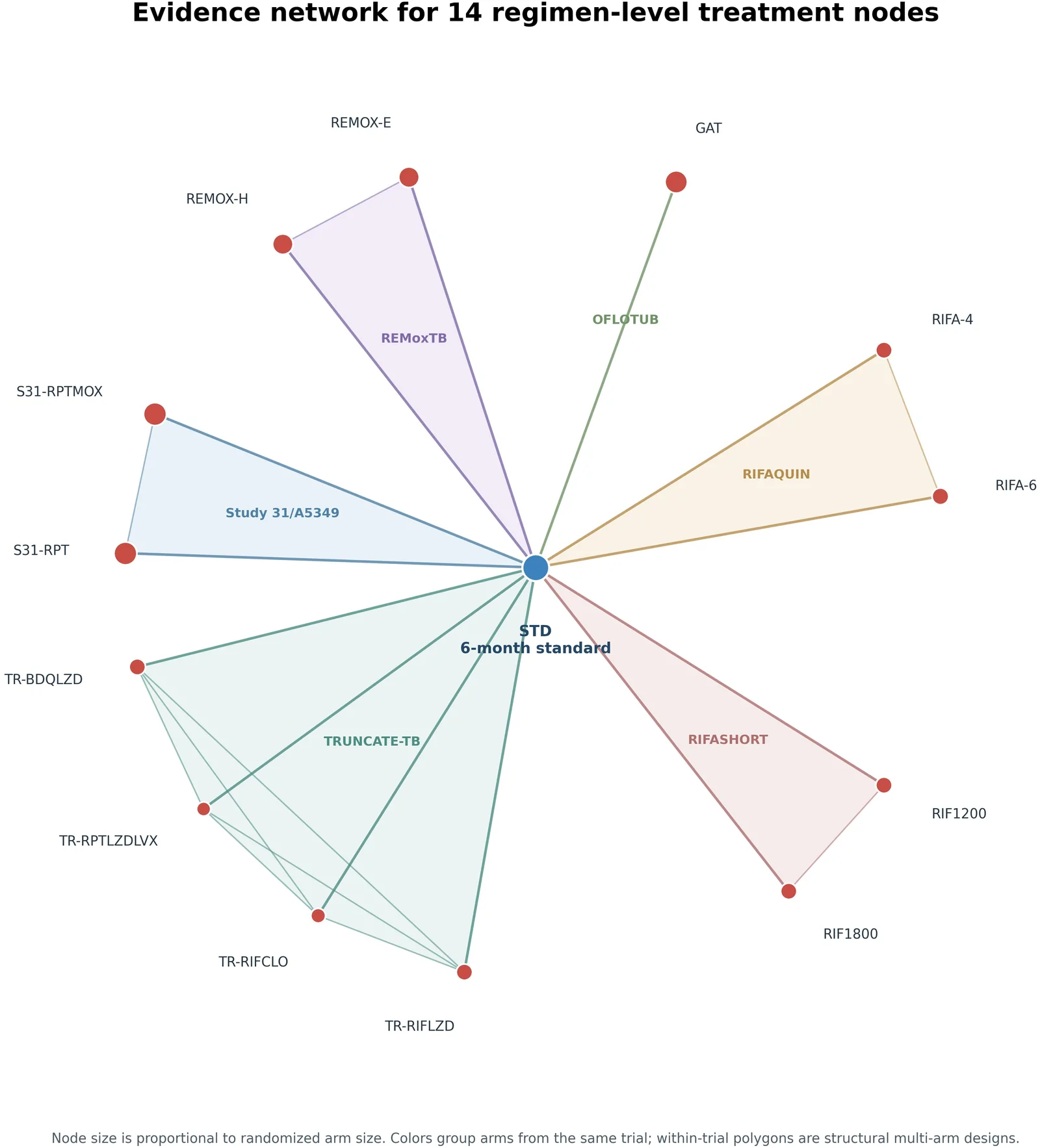
Overall network geometry for the 14 regimen-level treatment nodes included in the quantitative synthesis. The network was informed by six randomized controlled trials and contains 13 experimental-versus-standard edges plus 10 within-trial experimental-arm edges (23 unique edges in total). Five trials were multi-arm. Edge width is proportional to the number of direct comparisons and node size to randomized sample size. Abbreviations are defined in **Supplementary Table 3**.

Six of the eight included RCTs, involving 8,457 randomized participants, contributed to the NMA and informed 14 treatment nodes (**Figure 3**; **Supplementary Table 2**). The network comprised the daily 6-month standard reference, nine previously defined 4- or 6-month intervention nodes, and four intended 8-week TRUNCATE-TB nodes. Five trials were multi-arm. Across the network, there were 13 experimental-versus-standard edges and 10 within-trial experimental-arm edges, yielding 23 unique within-trial edges. Thirteen independent basic contrasts connected the 14 nodes.

All independent comparative evidence was anchored on the standard regimen, and the network contained no closed loop formed by independent trials. Structural loops within multi-arm trials did not permit independent inconsistency testing. A sensitivity network excluding TRUNCATE-TB yielded the same relative-effect estimates for the original ten nodes; adding TRUNCATE-TB changed the treatment set and therefore the descriptive P-scores, but not those original relative-effect estimates. Clinical transitivity was considered plausible for the mapped daily-control trials, although differences in disease severity, outcome definitions, follow-up, and TRUNCATE-TB’s response-guided implementation contributed to indirectness and were addressed in the certainty assessment.

### 3.4 NMA results

#### 3.4.1 Treatment success

The NMA included six RCTs and 14 treatment nodes (**Supplementary Table 3**). Treatment success was closest to the standard for the 6-month once-weekly rifapentine-moxifloxacin continuation regimen (RR = 1.01, 95% CI 0.93–1.09) and the Study 31/A5349 4-month daily rifapentine-moxifloxacin regimen (RR = 0.98, 95% CI 0.94–1.01). All intended 8-week TRUNCATE-TB regimens had lower treatment success than the standard: bedaquiline-linezolid RR = 0.84 (95% CI 0.77–0.91), high-dose rifampicin-clofazimine RR = 0.77 (95% CI 0.67–0.89), high-dose rifampicin-linezolid RR = 0.64 (95% CI 0.56–0.72), and rifapentine-linezolid-levofloxacin RR = 0.56 (95% CI 0.42–0.76). These are regimen-efficacy estimates and should not be interpreted as estimates of a surveillance-and-re-treatment management strategy.

#### 3.4.2 Relapse/recurrence-related outcomes

Relapse/recurrence-related outcomes with at least 12 months of follow-up were available from all six quantitative trials, although definitions and analysis populations differed (**Supplementary Table 3**). The 6-month once-weekly RIFAQUIN regimen had an imprecise estimate compatible with the standard (RR = 0.74, 95% CI 0.23–2.38), whereas the Study 31/A5349 4-month daily rifapentine-moxifloxacin regimen had RR = 1.82 (95% CI 1.12–2.96). Relapse/recurrence-related risk estimates were higher for every intended 8-week TRUNCATE-TB regimen: high-dose rifampicin-linezolid RR = 9.84 (95% CI 3.59–26.93), rifapentine-linezolid-levofloxacin RR = 6.46 (95% CI 1.91–21.89), high-dose rifampicin-clofazimine RR = 5.80 (95% CI 1.88–17.94), and bedaquiline-linezolid RR = 5.75 (95% CI 2.03–16.24). All four intended 8-week TRUNCATE-TB regimens yielded higher relapse/recurrence-related risk estimates than the standard regimen.

#### 3.4.3 Mortality

Mortality was uncommon and no comparison was statistically significant. RRs versus the standard ranged from 0.14 (95% CI 0.01–2.63) for the TRUNCATE-TB bedaquiline-linezolid regimen to 1.99 (95% CI 0.76–5.23) for the 4-month RIFAQUIN regimen. A 0.5 continuity correction was required for the TRUNCATE-TB mortality contrasts because two experimental arms had no deaths. The resulting intervals were wide; mortality estimates and rankings should therefore be interpreted cautiously and were not used to inform conclusions about regimen selection.

### 3.5 Ranking of treatment-shortening strategies

**Figure 4:**
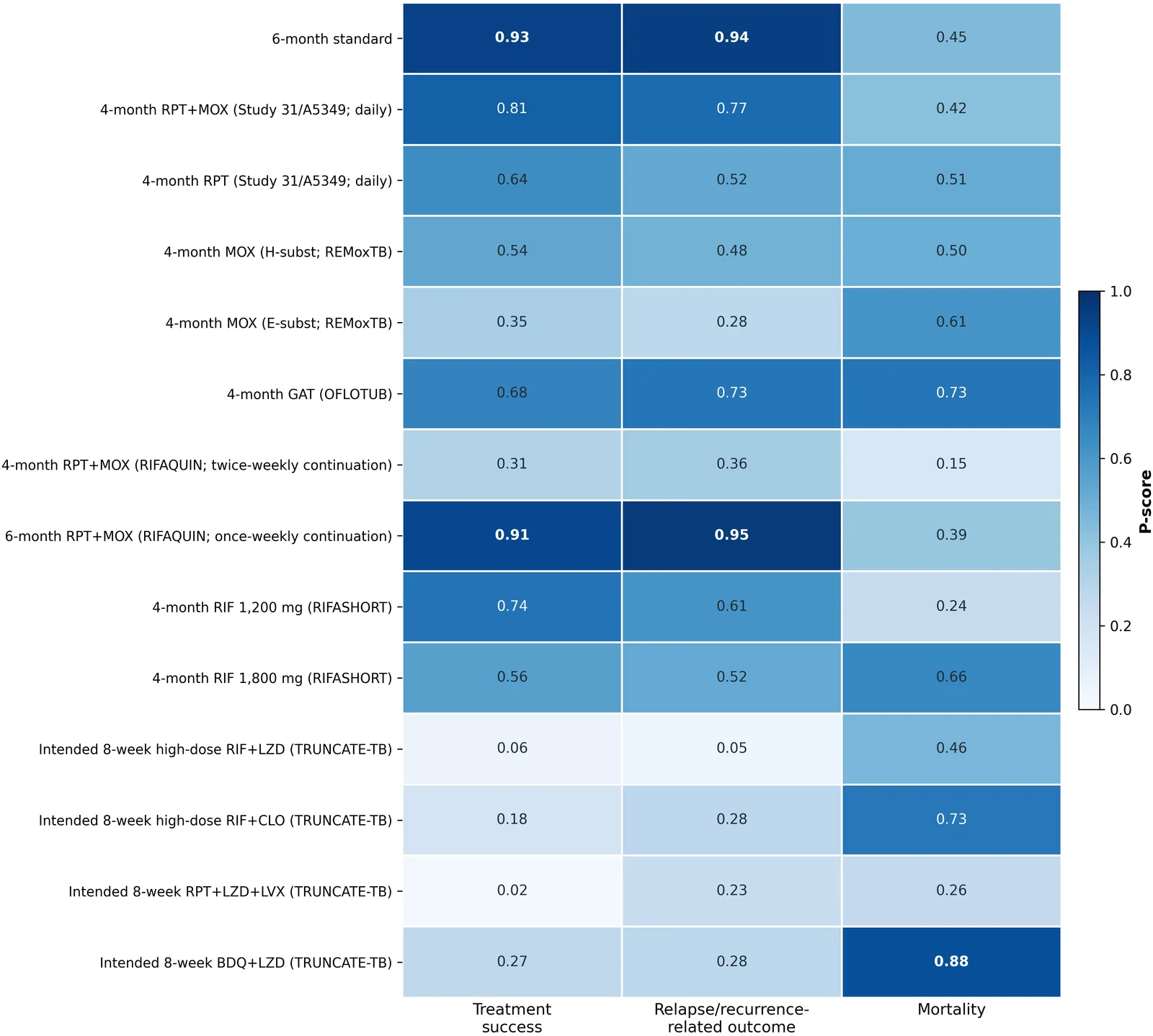
Heatmap of descriptive P-scores across outcomes for the 14 treatment nodes. Higher P-scores indicate higher treatment success and lower risks of relapse/recurrence-related outcomes or mortality. Rankings must be interpreted alongside relative-effect estimates and confidence intervals in **Supplementary Table 3**. Mortality rankings are particularly unstable because events were sparse and a continuity correction was required for TRUNCATE-TB.

Treatment strategies were ranked descriptively using P-scores (0–1), interpreted alongside **Supplementary Table 3** and not used to determine conclusions. For treatment success, the standard regimen (0.93) and the 6-month once-weekly RIFAQUIN regimen (0.91) ranked highest, whereas the four intended 8-week TRUNCATE-TB nodes ranked from 0.02 to 0.27. For relapse control, the 6-month RIFAQUIN regimen (0.95) and standard regimen (0.94) ranked highest; the TRUNCATE-TB nodes ranged from 0.05 to 0.28. Mortality P-scores ranged from 0.15 to 0.88 but were strongly influenced by sparse events and the continuity correction; they were therefore interpreted cautiously and not used to draw clinical conclusions. The composite heatmap (**Figure 4**) displays the same descriptive P-scores. Rankings varied by outcome and were considered subordinate to the relative-effect estimates and uncertainty in **Supplementary Table 3**.

### 3.6 Publication bias and small-study effects

Formal statistical assessment of funnel-plot asymmetry was not performed because fewer than 10 independent trials were available and several comparisons arose from multi-arm trials with shared comparator groups. The funnel plot is therefore presented for descriptive purposes only (**Supplementary Figure 1**), and publication bias or small-study effects could not be reliably assessed.

### 3.7 Risk of bias

Risk of bias was assessed with RoB 2 for the primary long-term efficacy result used from each of the eight RCTs. No trial was judged to be at high overall risk of bias; Study 31/A5349 and REMoxTB were rated low risk, and the remaining six trials had some concerns. Open-label status alone was not treated as sufficient for a judgement of some concerns. Rather, concerns were assigned when clinical or adjudicative components of a composite outcome could plausibly be influenced by knowledge of treatment assignment or when blinding of outcome assessors was insufficiently reported. Study 31/A5349 was rated low risk for outcome measurement because microbiologists and the central trial-operations team determining outcome status were unaware of treatment assignment and trial week. Additional concerns in some trials related to deviations from intended interventions, missing outcome data, or selection of the reported result. Domain-level assessments and the outcome-specific scope are presented in **Supplementary Figure 6**.

### 3.8 Certainty of evidence

Certainty of evidence was evaluated using the adapted CINeMA-style assessment described in Section 2.6 (**Supplementary Figure 7**). Certainty for treatment-success comparisons involving the standard regimen was generally low to moderate, whereas certainty for relapse and mortality was lower because of imprecision, outcome-definition differences, and sparse events. The TRUNCATE-TB nodes were informed by a single adaptive trial, with protocol-permitted treatment extension and small or early-stopped experimental arms contributing additional indirectness and imprecision. Heterogeneity was not assessable within the 14-node fixed-effect network, and incoherence was not applicable because there was no independent closed loop. Rankings were therefore treated as descriptive and subordinate to effect estimates and uncertainty.

## 4 Discussion

This NMA provides a comparative evaluation of treatment-shortening strategies for DS-PTB, with particular emphasis on treatment success and longer-term relapse/recurrence-related outcomes. The findings suggest that treatment duration alone does not fully account for long-term regimen performance, as clinically distinct regimens of similar duration yielded different estimates across these outcomes. The intended 8-week TRUNCATE-TB regimens were included in the primary quantitative synthesis and represented as regimen-level nodes in the NMA; in a pairwise sensitivity analysis, their exclusion did not change the direction of the pooled association, suggesting that the overall pattern was not solely attributable to their inclusion. At the same time, variation among individual regimens suggests that the pooled estimate should not be interpreted as indicating uniformly unfavorable effects across all shortened regimens. Taken together, these findings favor a regimen-specific rather than duration-only interpretation of treatment shortening and are consistent with the possibility that favorable early bacteriological responses may not always correspond to durable relapse control.

Regimen-specific findings further support this interpretation. In Study 31/A5349, the treatment-success estimate for the 4-month daily rifapentine-moxifloxacin regimen was close to that of the standard regimen, consistent with the non-inferiority conclusion of the original trial, whereas its recurrence-related estimate was less favorable. Because these analyses differed in endpoint definitions and analysis populations, this contrast should be interpreted cautiously; nevertheless, it illustrates why treatment success and post-treatment recurrence should not be regarded as interchangeable measures of long-term performance. The 6-month RIFAQUIN regimen with once-weekly rifapentine-moxifloxacin continuation produced estimates compatible with the standard regimen for both treatment success and recurrence, although the recurrence estimate was imprecise. The less favorable estimates for the 4-month RIFAQUIN rifapentine-moxifloxacin regimen further suggest that findings from one rifapentine-containing regimen should not necessarily be extrapolated to regimens with different dosing schedules or durations. Several 4-month fluoroquinolone-substitution regimens in REMoxTB and OFLOTUB likewise showed less favorable long-term outcomes despite early bacteriological activity reported in the original trials. This pattern is compatible with, but does not establish, a possible role for insufficient cumulative sterilizing activity against persistent bacillary populations (Barry et al., 2009; Mitchison and Davies, 2012; Wallis et al., 2000). The RIFASHORT findings likewise suggest that the effects of higher-dose rifampicin should be interpreted in the context of the complete regimen rather than as an isolated strategy for treatment shortening. Overall, the available evidence favors interpretation of the complete regimen rather than any single drug substitution, dose increase, or duration change in isolation.

TRUNCATE-TB warrants separate consideration because the present NMA incorporated the prespecified 2025 regimen-efficacy analysis of four intended 8-week regimens, whereas the earlier report evaluated a broader response-guided management strategy (Paton et al., 2023, Paton et al., 2025). Specifically, the four intended 8-week regimens were mapped to separate regimen-level nodes in the 14-node network. In the present analysis, these regimens showed lower treatment success and higher relapse/recurrence-related risk estimates than the 6-month standard regimen; however, these regimen-level estimates should not be equated with the effectiveness of the broader management strategy. That strategy also incorporated response-guided treatment extension, close post-treatment surveillance, and protocol-defined re-treatment, which may contribute to overall clinical outcomes. This distinction is clinically relevant because the feasibility of very short initial treatment may depend not only on the intended regimen itself but also on the capacity to identify patients who require treatment extension or subsequent re-treatment. Accordingly, the regimen-efficacy and management-strategy estimands provide complementary information and highlight the importance of considering the surrounding care pathway when very short treatment strategies are evaluated.

The clinical implications of these findings should therefore be considered at the level of specific regimens and patient populations rather than treatment duration alone. Evidence supporting a particular shortened regimen should not necessarily be extrapolated to other regimens of the same duration or containing the same drug class. The 4-month daily rifapentine-moxifloxacin regimen has been recommended for eligible patients under defined clinical and implementation conditions, and the present findings are consistent with interpreting the evidence at the level of the specific regimen rather than generalizing across all 4-month regimens (Carr et al., 2022; Saukkonen et al., 2025; World Health Organization, 2025). The findings also do not establish that intermittent dosing is inherently unfavorable; the 6-month once-weekly RIFAQUIN continuation regimen suggests that dosing frequency should be interpreted together with total treatment duration, companion-drug activity, and overall regimen design. Thus, the present analysis does not identify a universally optimal shortened regimen, but rather suggests that the balance between shorter treatment and durable outcomes may be regimen- and context-dependent.

Pharmacological and microbiological evidence provides a plausible framework for interpreting these regimen-level differences, although the underlying mechanisms cannot be established by the present NMA. Pharmacokinetic-pharmacodynamic studies have reported associations between rifamycin exposure, including the area under the concentration-time curve to minimum inhibitory concentration ratio (AUC/MIC), and post-treatment outcomes (Pasipanodya et al., 2013; Savic et al., 2017). Lesion-focused studies further indicate that heterogeneous drug penetration into relevant tissue compartments may influence sterilizing activity against persistent bacterial subpopulations (Dartois, 2014; Dartois and Rubin, 2022; Prideaux et al., 2015). These observations provide one possible framework for understanding why intensification of a single drug or a favorable early bacteriological response may not necessarily translate into durable relapse prevention, but they should not be regarded as mechanisms demonstrated by the present aggregate-data analysis (Mitchison and Davies, 2012). In this study, “long-term” referred to outcomes assessed ≥12 months after randomization or treatment completion, and the included trials generally reported follow-up periods of 12–24 months. This interval is clinically relevant because a substantial proportion of recurrent tuberculosis occurs within the first 12–18 months after treatment cessation (Imperial et al., 2018; Nahid et al., 2016). In higher-transmission settings, recurrent tuberculosis may reflect exogenous reinfection as well as endogenous relapse, and the relative contribution of reinfection may be greater than in lower-transmission settings (Vega et al., 2021, Vega et al., 2024). Because most included trials were conducted in moderate- or high-incidence regions across Africa, Asia, and Latin America and molecular typing was not consistently available, some recurrence events may therefore have represented reinfection rather than endogenous relapse. Recurrence/relapse-related estimates remain clinically relevant, but they should not necessarily be interpreted as pure measures of regimen-specific relapse prevention. Future treatment-shortening trials would benefit from incorporating molecular typing where feasible, particularly in high-burden settings.

Several limitations should be considered when interpreting these findings. Although eight randomized trials provided a geographically diverse evidence base and allowed comparison of a broad range of treatment-shortening strategies, six contributed to the quantitative synthesis, and most non-reference regimen nodes were informed by a single trial. The network contained no independent closed loop, so formal assessment of incoherence was not feasible, and the available network structure did not permit estimation of a common heterogeneity variance. Accordingly, relative-effect estimates and their confidence intervals should remain the primary basis for interpretation, with treatment rankings regarded as descriptive. Differences in eligibility criteria, disease severity, outcome definitions, analysis populations, follow-up schedules, and background transmission may also have affected comparability across trials. Recurrence-related endpoints were not defined uniformly, and some did not distinguish bacteriologically confirmed relapse from broader tuberculosis-related unfavorable outcomes. The TRUNCATE-TB regimen-level estimates were derived from a single adaptive trial conducted within a broader response-guided framework that permitted protocol-defined treatment extension, and some treatment arms were relatively small or stopped early; these features were considered in the assessment of indirectness and certainty. Two trials using thrice-weekly 6-month control regimens were retained in the systematic review and narrative synthesis but were not mapped to the quantitative network because these control regimens were not considered sufficiently exchangeable with the daily reference regimen. Mortality events were uncommon, and differences in adverse-event definitions limited quantitative synthesis of safety outcomes. Patient-centered and implementation outcomes, including treatment burden, preferences, quality of life, and cost-effectiveness, were also not consistently available across trials. In addition, the use of aggregate data restricted assessment of patient-level effect modifiers, adherence patterns, pharmacokinetic variability, and other factors that may influence suitability for treatment shortening. Future studies using harmonized long-term endpoints, molecular typing, individual-participant data, and patient-centered or implementation outcomes may further refine the interpretation and application of treatment-shortening strategies across clinical settings.

## 5 Conclusions

The standard 6-month regimen remains an established reference for durable treatment success in drug-susceptible pulmonary tuberculosis. Among shortened regimens, the 4-month daily rifapentine-moxifloxacin regimen in eligible patients showed a treatment-success estimate close to the standard regimen, whereas several other shortened or drug-substitution regimens showed less favorable long-term outcomes. Findings from TRUNCATE-TB further underscore the distinction between regimen-level efficacy and a broader management strategy that includes surveillance, treatment extension, and re-treatment. Overall, the evidence suggests that treatment-shortening strategies should be interpreted according to regimen composition, dosing, duration, patient selection, and implementation context rather than treatment duration alone.

## Data Availability

The original contributions presented in the study are included in the article/**Supplementary Material**. Further inquiries can be directed to the corresponding author.

## Supplementary material

Supplementary Figure 1 Funnel plot for the pairwise meta-analysis.

Supplementary Figure 2 Sensitivity analyses for the pairwise meta-analysis.

Supplementary Figure 3 Descriptive P-scores for treatment success.

Supplementary Figure 4 Descriptive P-scores for relapse/recurrence-related outcomes.

Supplementary Figure 5 Descriptive P-scores for mortality.

Supplementary Figure 6 Risk-of-bias assessment.

Supplementary Figure 7 Adapted CINeMA-style certainty assessment.

Methods S1 Literature search strategy and information sources.

Methods S2 Database-specific search strategies.

Methods S3 Study record management, duplicate detection, and screening.

Supplementary Table 1 Sensitivity analyses for the pairwise meta-analysis.

Supplementary Table 2 Study-arm-to-node mapping for all eligible randomized trials.

Supplementary Table 3 Outcome-specific data, fixed-effect network estimates, league tables, and descriptive P-scores.

Supplementary Table 4 Estimability of inconsistency assessments.
