# Supplementary material for: Treatment shortening and relapse control in drug-susceptible pulmonary tuberculosis: a network meta-analysis of randomized trials
Source: Front Cell Infect Microbiol. 2026 Sep 3;16:1869454. doi: 10.3389/fcimb.2026.1869454 (PMC13581796; doi:10.3389/fcimb.2026.1869454)
Supplement: Supplementary file 1 [file Supplementaryfile1.docx]

**Supplementary Material**

**Methods S1. Literature search strategy and information sources**

A systematic search was conducted from database inception to August 31, 2025, in MEDLINE (via PubMed), Embase, CENTRAL, Web of Science Core Collection, Scopus, CNKI, and Wanfang Data. ClinicalTrials.gov, the WHO ICTRP, reference lists of included studies and relevant reviews, conference abstracts, and academic theses were also screened. No language, publication-status, or publication-date restrictions were applied. Search results from Scopus, Wanfang Data, ClinicalTrials.gov, and WHO ICTRP were cross-checked against the master database but yielded no additional unique records for screening after cross-source deduplication. The source-specific identification information is reported in Figure 1.

The search used controlled vocabulary and free-text terms for drug-susceptible pulmonary tuberculosis, relevant treatment regimens, and randomized trials. Validated randomized-trial filters were applied in PubMed and Embase; no methodological filter was applied in CENTRAL, Web of Science, Scopus, CNKI, or Wanfang Data. Search results were exported on August 31, 2025, where export functions were available.
**Methods S2. Database-specific search strategies**

| **Source** | **Search strategy** |
| --- | --- |
| MEDLINE (PubMed) | ("Tuberculosis"[Mesh] OR tuberculosis[tiab] OR "pulmonary tuberculosis"[tiab]) AND ("drug susceptible"[tiab] OR "drug-susceptible"[tiab] OR "drug sensitive"[tiab] OR "drug-sensitive"[tiab]) AND (regimen*[tiab] OR treatment*[tiab] OR rifampicin[tiab] OR rifampin[tiab] OR rifapentine[tiab] OR moxifloxacin[tiab] OR gatifloxacin[tiab] OR bedaquiline[tiab] OR linezolid[tiab]) AND (randomized controlled trial[pt] OR controlled clinical trial[pt] OR random*[tiab] OR trial[tiab]) |
| Embase | ('pulmonary tuberculosis'/exp OR 'pulmonary tuberculosis':ti,ab OR tuberculosis:ti,ab) AND ('drug susceptible':ti,ab OR 'drug sensitive':ti,ab) AND (regimen*:ti,ab OR treatment*:ti,ab OR rifampicin:ti,ab OR rifapentine:ti,ab OR moxifloxacin:ti,ab OR gatifloxacin:ti,ab OR bedaquiline:ti,ab OR linezolid:ti,ab) AND ('randomized controlled trial'/exp OR random*:ti,ab OR trial:ti,ab) |
| CENTRAL | (tuberculosis OR "pulmonary tuberculosis") AND ("drug susceptible" OR "drug sensitive") AND (regimen* OR treatment* OR rifampicin OR rifapentine OR moxifloxacin OR gatifloxacin OR bedaquiline OR linezolid) |
| Web of Science Core Collection | TS=(tuberculosis OR "pulmonary tuberculosis") AND TS=("drug susceptible" OR "drug sensitive") AND TS=(regimen* OR treatment* OR rifampicin OR rifapentine OR moxifloxacin OR gatifloxacin OR bedaquiline OR linezolid) |
| Scopus | TITLE-ABS-KEY(tuberculosis OR "pulmonary tuberculosis") AND TITLE-ABS-KEY("drug susceptible" OR "drug sensitive") AND TITLE-ABS-KEY(regimen* OR treatment* OR rifampicin OR rifapentine OR moxifloxacin OR gatifloxacin OR bedaquiline OR linezolid) |
| CNKI | 主题=(肺结核 OR 结核病) AND 主题=(药物敏感 OR 敏感结核) AND 主题=(短程 OR 缩短疗程 OR 治疗方案 OR 利福平 OR 利福喷丁 OR 莫西沙星 OR 加替沙星) |
| Wanfang Data | 主题:(肺结核 OR 结核病) AND 主题:(药物敏感 OR 敏感结核) AND 主题:(短程 OR 缩短疗程 OR 治疗方案 OR 利福平 OR 利福喷丁 OR 莫西沙星 OR 加替沙星) |
| ClinicalTrials.gov / WHO ICTRP | Condition: pulmonary tuberculosis; Other terms: drug susceptible, treatment shortening, rifampicin, rifapentine, moxifloxacin, gatifloxacin, bedaquiline, or linezolid; Study type: interventional. |

Database syntax was adapted as required. Because database contents and indexing change over time, rerunning the strategies after the final search date may not reproduce the historical retrieval counts exactly.
**Methods S3. Study record management, duplicate detection, and screening**

All retrieved records were managed in a single EndNote library, supplemented by a structured spreadsheet. Records from sources supporting bulk citation export were exported in RIS or compatible formats and imported directly into EndNote. Records from sources without reference-manager-compatible bulk export, including trial registries, grey-literature sources, and hand-searched references, were entered manually into the spreadsheet, with the source, registry or accession identifier, and retrieval date recorded.

Duplicates were removed in two stages. First, automated detection was performed in EndNote based on Digital Object Identifier, title, author, journal, and publication year. Second, automatically flagged and near-duplicate records were manually verified, with uncertain matches checked against the original source. Multiple reports of the same trial, including primary reports, extended follow-up reports, or secondary analyses, were linked to a single study record rather than counted as separate studies. Record management and deduplication govern how studies were counted; they are distinct from the analytical handling of multi-arm trials, the assessment of transitivity, and the assessment of consistency, which are described in Sections 2.4 and 2.5 of the main text and are summarized numerically in Tables S2 and S4.

After deduplication, two reviewers independently screened titles and abstracts, followed by full-text assessment. Full texts were retrieved only for records considered potentially eligible after title and abstract screening, rather than for every retrieved record at the initial stage. Disagreements were resolved through discussion, with consultation of a third reviewer when necessary. Reasons for exclusion at the full-text screening stage were documented and are presented in the PRISMA flow diagram.

**Figure S1. Funnel plot for the pairwise meta-analysis of treatment-shortening regimens versus the 6-month standard regimen.**

The funnel plot is presented for descriptive purposes only. Formal statistical assessment of funnel-plot asymmetry was not performed because fewer than 10 independent source trials were available and several comparisons arose from multi-arm trials. The four TRUNCATE-TB points share a common control and should not be interpreted as four independent studies.

**Table S1. Sensitivity analyses for the pairwise meta-analysis**

| **Analysis** | **Estimator** | **Source trials** | **RR** | **95% CI** | **I²** |
| --- | --- | --- | --- | --- | --- |
| Primary | Random effects (DL) | 6 | 1.68 | 1.37–2.06 | 39.9% |
| Fixed-effect sensitivity | Inverse variance | 6 | 1.52 | 1.33–1.74 | — |
| HKSJ sensitivity | Random effects (HKSJ) | 6 | 1.68 | 1.34–2.10 | 39.9% |
| Excluding TRUNCATE-TB | Random effects (DL) | 5 | 1.54 | 1.29–1.85 | 33.7% |

Twelve comparisons from six randomized controlled trials contributed. Outcome data from 6,752 participants, including 4,298 participants in shortened-regimen groups and 2,454 comparator participants, informed the primary pairwise estimate. Shared comparator groups were divided equally for weighting while original control risks were preserved.

**Table S2. Study-arm-to-node mapping for the eight eligible randomized controlled trials**

Six source trials (8,457 randomized participants) informed the 14-node NMA; outcome data from 6,752 participants contributed to the pooled pairwise estimate. Two trials (1,787 participants) were retained for narrative synthesis only because their thrice-weekly control regimens were not exchangeable with the daily reference node.

| **Study** | **Randomized arm** | **Treatment node** | **Quantitative contribution** |
| --- | --- | --- | --- |
| Dorman et al., 2021 | 4-month daily RPT+MOX | S31-RPTMOX | Included |
|  | 4-month daily RPT | S31-RPT | Included |
|  | 6-month daily standard | STD | Reference |
| Gillespie et al., 2014 | 4-month MOX (H substitution) | REMOX-H | Included |
|  | 4-month MOX (E substitution) | REMOX-E | Included |
|  | 6-month daily standard | STD | Reference |
| Merle et al., 2014 | 4-month GAT | GAT | Included |
|  | 6-month daily standard | STD | Reference |
| Jindani et al., 2014 | 4-month RPT+MOX; twice-weekly continuation | RIFA-4 | Included |
|  | 6-month RPT+MOX; once-weekly continuation | RIFA-6 | Included in NMA; not in shortened-duration pairwise pool |
|  | 6-month daily standard | STD | Reference |
| Jindani et al., 2023 | 4-month RIF 1,200 mg | RIF1200 | Included |
|  | 4-month RIF 1,800 mg | RIF1800 | Included |
|  | 6-month daily standard | STD | Reference |
| Paton et al., 2025 | Intended 8-week high-dose RIF+LZD (n=184) | TR-RIFLZD | Included; prespecified regimen-efficacy analysis |
|  | Intended 8-week high-dose RIF+CLO (n=78) | TR-RIFCLO | Included; prespecified regimen-efficacy analysis |
|  | Intended 8-week RPT+LZD+LVX (n=42) | TR-RPTLZDLVX | Included; prespecified regimen-efficacy analysis |
|  | Intended 8-week BDQ+LZD (n=189) | TR-BDQLZD | Included; prespecified regimen-efficacy analysis |
|  | 24-week daily standard (n=181) | STD | Reference; shared control |
| Jawahar et al., 2013 | 4-month thrice-weekly MOX regimen | Not mapped | Narrative only: thrice-weekly standard control |
|  | 4-month thrice-weekly GAT regimen | Not mapped | Narrative only: thrice-weekly standard control |
|  | 6-month thrice-weekly control | Not mapped | Not exchangeable with daily STD |
| Velayutham et al., 2020 | M3: 3-month daily MOX regimen | Not mapped | Narrative only: thrice-weekly standard control |
|  | M4: 4-month daily MOX regimen | Not mapped | Narrative only: thrice-weekly standard control |
|  | M4-I: 4-month regimen; intermittent continuation | Not mapped | Narrative only: thrice-weekly standard control |
|  | M4-IE: 4-month regimen; intermittent continuation | Not mapped | Narrative only: thrice-weekly standard control |
|  | 6-month thrice-weekly control | Not mapped | Not exchangeable with daily STD |

**Table S3. Fixed-effect network meta-analysis estimates and descriptive P-scores**

The table reports the 14-node consistency model used in the final analysis. Risk ratios (RRs) above 1 favor the row treatment for treatment success and favor the column treatment for relapse/recurrence-related outcomes and mortality. All league-table cells report the RR for the row treatment versus the column treatment.

**Panel A. Outcome-specific source data and estimates versus the 6-month standard regimen**

| **Outcome** | **Treatment node** | **Trial** | **Events/n** | **Standard events/n** | **RR versus standard (95% CI)** | **P-score** | **Direction** | **Notes** |
| --- | --- | --- | --- | --- | --- | --- | --- | --- |
| Treatment success | 6-month standard | Common reference | See comparator columns | Reference | 1.00 (reference) | 0.93 | Higher favorable |  |
| Treatment success | 4-month RPT+MOX (Study 31/A5349; daily) | Study 31/A5349 | 668/756 | 656/726 | 0.98 (0.94–1.01) | 0.81 | Higher favorable | Trial-reported analysis population |
| Treatment success | 4-month RPT (Study 31/A5349; daily) | Study 31/A5349 | 645/752 | 656/726 | 0.95 (0.91–0.99) | 0.64 | Higher favorable | Trial-reported analysis population |
| Treatment success | 4-month MOX (H-subst; REMoxTB) | REMoxTB | 436/514 | 467/510 | 0.93 (0.89–0.97) | 0.54 | Higher favorable | Trial-reported analysis population |
| Treatment success | 4-month MOX (E-subst; REMoxTB) | REMoxTB | 419/524 | 467/510 | 0.87 (0.83–0.92) | 0.35 | Higher favorable | Trial-reported analysis population |
| Treatment success | 4-month GAT (OFLOTUB) | OFLOTUB | 548/694 | 548/662 | 0.95 (0.91–1.00) | 0.68 | Higher favorable | Trial-reported analysis population |
| Treatment success | 4-month RPT+MOX (RIFAQUIN; twice-weekly continuation) | RIFAQUIN | 141/193 | 161/188 | 0.85 (0.77–0.95) | 0.31 | Higher favorable | Trial-reported analysis population |
| Treatment success | 6-month RPT+MOX (RIFAQUIN; once-weekly continuation) | RIFAQUIN | 183/212 | 161/188 | 1.01 (0.93–1.09) | 0.91 | Higher favorable | Trial-reported analysis population |
| Treatment success | 4-month RIF 1,200 mg (RIFASHORT) | RIFASHORT | 167/186 | 174/187 | 0.96 (0.91–1.03) | 0.74 | Higher favorable | Trial-reported analysis population |
| Treatment success | 4-month RIF 1,800 mg (RIFASHORT) | RIFASHORT | 161/186 | 174/187 | 0.93 (0.87–1.00) | 0.56 | Higher favorable | Trial-reported analysis population |
| Treatment success | Intended 8-week high-dose RIF+LZD (TRUNCATE-TB) | TRUNCATE-TB | 109/184 | 168/181 | 0.64 (0.56–0.72) | 0.06 | Higher favorable | ITT; prespecified regimen-level analysis |
| Treatment success | Intended 8-week high-dose RIF+CLO (TRUNCATE-TB) | TRUNCATE-TB | 56/78 | 168/181 | 0.77 (0.67–0.89) | 0.18 | Higher favorable | ITT; prespecified regimen-level analysis |
| Treatment success | Intended 8-week RPT+LZD+LVX (TRUNCATE-TB) | TRUNCATE-TB | 22/42 | 168/181 | 0.56 (0.42–0.76) | 0.02 | Higher favorable | ITT; prespecified regimen-level analysis |
| Treatment success | Intended 8-week BDQ+LZD (TRUNCATE-TB) | TRUNCATE-TB | 147/189 | 168/181 | 0.84 (0.77–0.91) | 0.27 | Higher favorable | ITT; prespecified regimen-level analysis |
| Relapse/recurrence-related outcome | 6-month standard | Common reference | See comparator columns | Reference | 1.00 (reference) | 0.94 | Lower favorable |  |
| Relapse/recurrence-related outcome | 4-month RPT+MOX (Study 31/A5349; daily) | Study 31/A5349 | 45/791 | 24/768 | 1.82 (1.12–2.96) | 0.77 | Lower favorable | Trial-reported analysis population |
| Relapse/recurrence-related outcome | 4-month RPT (Study 31/A5349; daily) | Study 31/A5349 | 75/784 | 24/768 | 3.06 (1.95–4.80) | 0.52 | Lower favorable | Trial-reported analysis population |
| Relapse/recurrence-related outcome | 4-month MOX (H-subst; REMoxTB) | REMoxTB | 46/568 | 13/555 | 3.46 (1.89–6.33) | 0.48 | Lower favorable | Trial-reported analysis population |
| Relapse/recurrence-related outcome | 4-month MOX (E-subst; REMoxTB) | REMoxTB | 64/551 | 13/555 | 4.96 (2.76–8.90) | 0.28 | Lower favorable | Trial-reported analysis population |
| Relapse/recurrence-related outcome | 4-month GAT (OFLOTUB) | OFLOTUB | 101/694 | 47/662 | 2.05 (1.47–2.85) | 0.73 | Lower favorable | Trial-reported analysis population |
| Relapse/recurrence-related outcome | 4-month RPT+MOX (RIFAQUIN; twice-weekly continuation) | RIFAQUIN | 27/193 | 6/188 | 4.38 (1.85–10.37) | 0.36 | Lower favorable | Trial-reported analysis population |
| Relapse/recurrence-related outcome | 6-month RPT+MOX (RIFAQUIN; once-weekly continuation) | RIFAQUIN | 5/212 | 6/188 | 0.74 (0.23–2.38) | 0.95 | Lower favorable | Trial-reported analysis population |
| Relapse/recurrence-related outcome | 4-month RIF 1,200 mg (RIFASHORT) | RIFASHORT | 10/186 | 4/187 | 2.51 (0.80–7.87) | 0.61 | Lower favorable | Trial-reported analysis population |
| Relapse/recurrence-related outcome | 4-month RIF 1,800 mg (RIFASHORT) | RIFASHORT | 12/186 | 4/187 | 3.02 (0.99–9.18) | 0.52 | Lower favorable | Trial-reported analysis population |
| Relapse/recurrence-related outcome | Intended 8-week high-dose RIF+LZD (TRUNCATE-TB) | TRUNCATE-TB | 40/184 | 4/181 | 9.84 (3.59–26.93) | 0.05 | Lower favorable | ITT; prespecified regimen-level analysis |
| Relapse/recurrence-related outcome | Intended 8-week high-dose RIF+CLO (TRUNCATE-TB) | TRUNCATE-TB | 10/78 | 4/181 | 5.80 (1.88–17.94) | 0.28 | Lower favorable | ITT; prespecified regimen-level analysis |
| Relapse/recurrence-related outcome | Intended 8-week RPT+LZD+LVX (TRUNCATE-TB) | TRUNCATE-TB | 6/42 | 4/181 | 6.46 (1.91–21.89) | 0.23 | Lower favorable | ITT; prespecified regimen-level analysis |
| Relapse/recurrence-related outcome | Intended 8-week BDQ+LZD (TRUNCATE-TB) | TRUNCATE-TB | 24/189 | 4/181 | 5.75 (2.03–16.24) | 0.28 | Lower favorable | ITT; prespecified regimen-level analysis |
| All-cause mortality | 6-month standard | Common reference | See comparator columns | Reference | 1.00 (reference) | 0.45 | Lower favorable |  |
| All-cause mortality | 4-month RPT+MOX (Study 31/A5349; daily) | Study 31/A5349 | 13/846 | 12/825 | 1.06 (0.48–2.30) | 0.42 | Lower favorable | Trial-reported analysis population |
| All-cause mortality | 4-month RPT (Study 31/A5349; daily) | Study 31/A5349 | 11/835 | 12/825 | 0.91 (0.40–2.04) | 0.51 | Lower favorable | Trial-reported analysis population |
| All-cause mortality | 4-month MOX (H-subst; REMoxTB) | REMoxTB | 15/655 | 16/639 | 0.91 (0.46–1.83) | 0.50 | Lower favorable | Trial-reported analysis population |
| All-cause mortality | 4-month MOX (E-subst; REMoxTB) | REMoxTB | 12/636 | 16/639 | 0.75 (0.36–1.58) | 0.61 | Lower favorable | Trial-reported analysis population |
| All-cause mortality | 4-month GAT (OFLOTUB) | OFLOTUB | 5/694 | 9/662 | 0.53 (0.18–1.57) | 0.73 | Lower favorable | Trial-reported analysis population |
| All-cause mortality | 4-month RPT+MOX (RIFAQUIN; twice-weekly continuation) | RIFAQUIN | 12/276 | 6/275 | 1.99 (0.76–5.23) | 0.15 | Lower favorable | Trial-reported analysis population |
| All-cause mortality | 6-month RPT+MOX (RIFAQUIN; once-weekly continuation) | RIFAQUIN | 7/276 | 6/275 | 1.16 (0.40–3.41) | 0.39 | Lower favorable | Trial-reported analysis population |
| All-cause mortality | 4-month RIF 1,200 mg (RIFASHORT) | RIFASHORT | 8/223 | 5/224 | 1.61 (0.53–4.84) | 0.24 | Lower favorable | Trial-reported analysis population |
| All-cause mortality | 4-month RIF 1,800 mg (RIFASHORT) | RIFASHORT | 3/225 | 5/224 | 0.60 (0.14–2.47) | 0.66 | Lower favorable | Trial-reported analysis population |
| All-cause mortality | Intended 8-week high-dose RIF+LZD (TRUNCATE-TB) | TRUNCATE-TB | 3/184 | 3/181 | 0.98 (0.23–4.27) | 0.46 | Lower favorable | 0.5 continuity correction used for all TRUNCATE-TB arms in mortality model |
| All-cause mortality | Intended 8-week high-dose RIF+CLO (TRUNCATE-TB) | TRUNCATE-TB | 0/78 | 3/181 | 0.33 (0.02–6.30) | 0.73 | Lower favorable | 0.5 continuity correction used for all TRUNCATE-TB arms in mortality model |
| All-cause mortality | Intended 8-week RPT+LZD+LVX (TRUNCATE-TB) | TRUNCATE-TB | 1/42 | 3/181 | 1.81 (0.28–11.93) | 0.26 | Lower favorable | 0.5 continuity correction used for all TRUNCATE-TB arms in mortality model |
| All-cause mortality | Intended 8-week BDQ+LZD (TRUNCATE-TB) | TRUNCATE-TB | 0/189 | 3/181 | 0.14 (0.01–2.63) | 0.88 | Lower favorable | 0.5 continuity correction used for all TRUNCATE-TB arms in mortality model |

Outcome definitions and denominators were outcome specific. Treatment success used each trial's primary efficacy or assessable population. Relapse/recurrence-related outcomes used post-treatment recurrence or relapse where reported; Study 31/A5349 contributed tuberculosis-related unfavorable outcomes rather than pure relapse, and RIFASHORT recurrence included bacteriological or clinically adjudicated recurrence. TRUNCATE-TB used the prespecified 2025 regimen-efficacy ITT analysis, distinct from the earlier management-strategy estimand. Mortality used all-cause deaths in the broadest reported randomized or safety population, except OFLOTUB reported deaths during treatment. These differences were considered in the transitivity and certainty assessments.

**Panel B. League table for Treatment success**

| **Row treatment** | **1. STD** | **2. S31-RPTMOX** | **3. S31-RPT** | **4. REMOX-H** | **5. REMOX-E** | **6. GAT** | **7. RIFA-4** | **8. RIFA-6** | **9. RIF1200** | **10. RIF1800** | **11. TR-RIFLZD** | **12. TR-RIFCLO** | **13. TR-RPTLZDLVX** | **14. TR-BDQLZD** |
| --- | --- | --- | --- | --- | --- | --- | --- | --- | --- | --- | --- | --- | --- | --- |
| 1. STD | 1.00 (self-comparison) | 1.02 (0.99–1.06) | 1.05 (1.01–1.09) | 1.08 (1.03–1.13) | 1.15 (1.09–1.20) | 1.05 (1.00–1.10) | 1.17 (1.06–1.30) | 0.99 (0.92–1.07) | 1.04 (0.97–1.10) | 1.07 (1.00–1.15) | 1.57 (1.38–1.78) | 1.29 (1.12–1.49) | 1.77 (1.32–2.37) | 1.19 (1.09–1.30) |
| 2. S31-RPTMOX | 0.98 (0.94–1.01) | 1.00 (self-comparison) | 1.03 (0.99–1.07) | 1.06 (1.00–1.12) | 1.12 (1.05–1.19) | 1.03 (0.96–1.09) | 1.15 (1.03–1.28) | 0.97 (0.89–1.06) | 1.01 (0.94–1.09) | 1.05 (0.97–1.14) | 1.53 (1.34–1.75) | 1.26 (1.09–1.47) | 1.73 (1.29–2.32) | 1.17 (1.06–1.28) |
| 3. S31-RPT | 0.95 (0.91–0.99) | 0.97 (0.93–1.01) | 1.00 (self-comparison) | 1.02 (0.97–1.09) | 1.09 (1.02–1.16) | 1.00 (0.93–1.06) | 1.11 (1.00–1.24) | 0.94 (0.86–1.03) | 0.98 (0.91–1.06) | 1.02 (0.94–1.10) | 1.49 (1.30–1.70) | 1.23 (1.06–1.43) | 1.68 (1.25–2.26) | 1.13 (1.03–1.24) |
| 4. REMOX-H | 0.93 (0.89–0.97) | 0.95 (0.89–1.00) | 0.98 (0.92–1.03) | 1.00 (self-comparison) | 1.06 (1.00–1.12) | 0.97 (0.91–1.04) | 1.09 (0.97–1.22) | 0.92 (0.84–1.01) | 0.96 (0.89–1.04) | 1.00 (0.92–1.08) | 1.45 (1.27–1.66) | 1.20 (1.03–1.39) | 1.64 (1.22–2.20) | 1.11 (1.00–1.22) |
| 5. REMOX-E | 0.87 (0.83–0.92) | 0.89 (0.84–0.95) | 0.92 (0.86–0.98) | 0.94 (0.89–1.00) | 1.00 (self-comparison) | 0.92 (0.85–0.98) | 1.02 (0.91–1.15) | 0.87 (0.79–0.95) | 0.90 (0.84–0.98) | 0.94 (0.86–1.02) | 1.37 (1.19–1.57) | 1.13 (0.97–1.32) | 1.55 (1.15–2.08) | 1.04 (0.94–1.15) |
| 6. GAT | 0.95 (0.91–1.00) | 0.98 (0.92–1.04) | 1.00 (0.94–1.07) | 1.03 (0.96–1.10) | 1.09 (1.02–1.17) | 1.00 (self-comparison) | 1.12 (1.00–1.26) | 0.95 (0.86–1.04) | 0.99 (0.91–1.07) | 1.03 (0.94–1.12) | 1.49 (1.30–1.71) | 1.23 (1.06–1.44) | 1.69 (1.26–2.27) | 1.14 (1.03–1.26) |
| 7. RIFA-4 | 0.85 (0.77–0.95) | 0.87 (0.78–0.97) | 0.90 (0.80–1.00) | 0.92 (0.82–1.03) | 0.98 (0.87–1.10) | 0.89 (0.80–1.00) | 1.00 (self-comparison) | 0.85 (0.76–0.94) | 0.88 (0.78–1.00) | 0.92 (0.81–1.04) | 1.34 (1.13–1.57) | 1.10 (0.92–1.32) | 1.51 (1.11–2.06) | 1.02 (0.89–1.17) |
| 8. RIFA-6 | 1.01 (0.93–1.09) | 1.03 (0.95–1.12) | 1.06 (0.97–1.16) | 1.09 (0.99–1.19) | 1.15 (1.05–1.27) | 1.06 (0.96–1.16) | 1.18 (1.07–1.31) | 1.00 (self-comparison) | 1.04 (0.94–1.16) | 1.08 (0.98–1.20) | 1.58 (1.36–1.83) | 1.30 (1.10–1.54) | 1.79 (1.32–2.42) | 1.20 (1.07–1.35) |
| 9. RIF1200 | 0.96 (0.91–1.03) | 0.99 (0.92–1.06) | 1.02 (0.95–1.09) | 1.04 (0.96–1.12) | 1.10 (1.02–1.20) | 1.01 (0.93–1.10) | 1.13 (1.00–1.28) | 0.96 (0.87–1.06) | 1.00 (self-comparison) | 1.04 (0.96–1.12) | 1.51 (1.31–1.74) | 1.25 (1.07–1.46) | 1.71 (1.27–2.30) | 1.15 (1.04–1.28) |
| 10. RIF1800 | 0.93 (0.87–1.00) | 0.95 (0.88–1.03) | 0.98 (0.91–1.06) | 1.00 (0.92–1.09) | 1.07 (0.98–1.16) | 0.98 (0.89–1.06) | 1.09 (0.96–1.24) | 0.92 (0.83–1.03) | 0.96 (0.89–1.04) | 1.00 (self-comparison) | 1.46 (1.26–1.68) | 1.20 (1.02–1.41) | 1.65 (1.22–2.22) | 1.11 (0.99–1.24) |
| 11. TR-RIFLZD | 0.64 (0.56–0.72) | 0.65 (0.57–0.74) | 0.67 (0.59–0.77) | 0.69 (0.60–0.79) | 0.73 (0.64–0.84) | 0.67 (0.58–0.77) | 0.75 (0.64–0.88) | 0.63 (0.55–0.74) | 0.66 (0.57–0.76) | 0.69 (0.59–0.79) | 1.00 (self-comparison) | 0.83 (0.69–0.99) | 1.13 (0.83–1.55) | 0.76 (0.66–0.88) |
| 12. TR-RIFCLO | 0.77 (0.67–0.89) | 0.79 (0.68–0.92) | 0.81 (0.70–0.95) | 0.83 (0.72–0.97) | 0.89 (0.76–1.03) | 0.81 (0.70–0.95) | 0.91 (0.76–1.08) | 0.77 (0.65–0.91) | 0.80 (0.68–0.94) | 0.83 (0.71–0.98) | 1.21 (1.01–1.46) | 1.00 (self-comparison) | 1.37 (1.00–1.89) | 0.92 (0.79–1.08) |
| 13. TR-RPTLZDLVX | 0.56 (0.42–0.76) | 0.58 (0.43–0.77) | 0.59 (0.44–0.80) | 0.61 (0.45–0.82) | 0.65 (0.48–0.87) | 0.59 (0.44–0.80) | 0.66 (0.49–0.90) | 0.56 (0.41–0.76) | 0.58 (0.43–0.79) | 0.61 (0.45–0.82) | 0.88 (0.65–1.21) | 0.73 (0.53–1.00) | 1.00 (self-comparison) | 0.67 (0.50–0.91) |
| 14. TR-BDQLZD | 0.84 (0.77–0.91) | 0.86 (0.78–0.94) | 0.88 (0.80–0.97) | 0.90 (0.82–1.00) | 0.96 (0.87–1.06) | 0.88 (0.79–0.97) | 0.98 (0.86–1.12) | 0.83 (0.74–0.93) | 0.87 (0.78–0.97) | 0.90 (0.81–1.01) | 1.31 (1.14–1.51) | 1.08 (0.92–1.27) | 1.48 (1.10–2.00) | 1.00 (self-comparison) |

Node key: 1. STD = 6-month standard; 2. S31-RPTMOX = 4-month RPT+MOX (Study 31/A5349; daily); 3. S31-RPT = 4-month RPT (Study 31/A5349; daily); 4. REMOX-H = 4-month MOX (H-subst; REMoxTB); 5. REMOX-E = 4-month MOX (E-subst; REMoxTB); 6. GAT = 4-month GAT (OFLOTUB); 7. RIFA-4 = 4-month RPT+MOX (RIFAQUIN; twice-weekly continuation); 8. RIFA-6 = 6-month RPT+MOX (RIFAQUIN; once-weekly continuation); 9. RIF1200 = 4-month RIF 1,200 mg (RIFASHORT); 10. RIF1800 = 4-month RIF 1,800 mg (RIFASHORT); 11. TR-RIFLZD = intended 8-week high-dose RIF+LZD; 12. TR-RIFCLO = intended 8-week high-dose RIF+CLO; 13. TR-RPTLZDLVX = intended 8-week RPT+LZD+LVX; 14. TR-BDQLZD = intended 8-week BDQ+LZD.

Model: frequentist fixed-effect consistency NMA on the log-risk-ratio scale. Correlations induced by shared comparator arms in Study 31/A5349, REMoxTB, RIFAQUIN, RIFASHORT, and TRUNCATE-TB were retained through common-control covariance. The 14-node network had 13 independent basic contrasts and no residual degrees of freedom for estimating a network heterogeneity variance. No continuity correction was required for treatment success.

**Panel C. League table for Relapse/recurrence-related outcome**

| **Row treatment** | **1. STD** | **2. S31-RPTMOX** | **3. S31-RPT** | **4. REMOX-H** | **5. REMOX-E** | **6. GAT** | **7. RIFA-4** | **8. RIFA-6** | **9. RIF1200** | **10. RIF1800** | **11. TR-RIFLZD** | **12. TR-RIFCLO** | **13. TR-RPTLZDLVX** | **14. TR-BDQLZD** |
| --- | --- | --- | --- | --- | --- | --- | --- | --- | --- | --- | --- | --- | --- | --- |
| 1. STD | 1.00 (self-comparison) | 0.55 (0.34–0.89) | 0.33 (0.21–0.51) | 0.29 (0.16–0.53) | 0.20 (0.11–0.36) | 0.49 (0.35–0.68) | 0.23 (0.10–0.54) | 1.35 (0.42–4.36) | 0.40 (0.13–1.25) | 0.33 (0.11–1.01) | 0.10 (0.04–0.28) | 0.17 (0.06–0.53) | 0.15 (0.05–0.52) | 0.17 (0.06–0.49) |
| 2. S31-RPTMOX | 1.82 (1.12–2.96) | 1.00 (self-comparison) | 0.59 (0.42–0.85) | 0.53 (0.24–1.14) | 0.37 (0.17–0.78) | 0.89 (0.49–1.60) | 0.42 (0.15–1.12) | 2.46 (0.69–8.75) | 0.72 (0.21–2.50) | 0.60 (0.18–2.03) | 0.19 (0.06–0.57) | 0.31 (0.09–1.07) | 0.28 (0.08–1.05) | 0.32 (0.10–1.00) |
| 3. S31-RPT | 3.06 (1.95–4.80) | 1.68 (1.18–2.40) | 1.00 (self-comparison) | 0.89 (0.42–1.88) | 0.62 (0.30–1.29) | 1.49 (0.86–2.61) | 0.70 (0.26–1.84) | 4.14 (1.18–14.51) | 1.22 (0.36–4.15) | 1.01 (0.31–3.37) | 0.31 (0.10–0.94) | 0.53 (0.16–1.78) | 0.47 (0.13–1.74) | 0.53 (0.17–1.65) |
| 4. REMOX-H | 3.46 (1.89–6.33) | 1.90 (0.87–4.12) | 1.13 (0.53–2.40) | 1.00 (self-comparison) | 0.70 (0.49–1.00) | 1.69 (0.85–3.36) | 0.79 (0.28–2.26) | 4.68 (1.25–17.47) | 1.38 (0.38–5.01) | 1.15 (0.32–4.07) | 0.35 (0.11–1.14) | 0.60 (0.17–2.14) | 0.53 (0.14–2.09) | 0.60 (0.18–2.00) |
| 5. REMOX-E | 4.96 (2.76–8.90) | 2.72 (1.27–5.82) | 1.62 (0.78–3.38) | 1.43 (1.00–2.06) | 1.00 (self-comparison) | 2.42 (1.24–4.73) | 1.13 (0.40–3.20) | 6.71 (1.81–24.83) | 1.97 (0.55–7.11) | 1.64 (0.47–5.78) | 0.50 (0.16–1.62) | 0.85 (0.24–3.05) | 0.77 (0.20–2.97) | 0.86 (0.26–2.84) |
| 6. GAT | 2.05 (1.47–2.85) | 1.13 (0.63–2.02) | 0.67 (0.38–1.17) | 0.59 (0.30–1.18) | 0.41 (0.21–0.81) | 1.00 (self-comparison) | 0.47 (0.19–1.18) | 2.77 (0.82–9.36) | 0.82 (0.25–2.68) | 0.68 (0.21–2.17) | 0.21 (0.07–0.60) | 0.35 (0.11–1.15) | 0.32 (0.09–1.12) | 0.36 (0.12–1.06) |
| 7. RIFA-4 | 4.38 (1.85–10.37) | 2.41 (0.90–6.47) | 1.43 (0.54–3.78) | 1.27 (0.44–3.63) | 0.88 (0.31–2.50) | 2.14 (0.85–5.38) | 1.00 (self-comparison) | 5.93 (2.33–15.10) | 1.74 (0.42–7.29) | 1.45 (0.36–5.94) | 0.45 (0.12–1.68) | 0.76 (0.18–3.13) | 0.68 (0.15–3.02) | 0.76 (0.20–2.94) |
| 8. RIFA-6 | 0.74 (0.23–2.38) | 0.41 (0.11–1.44) | 0.24 (0.07–0.85) | 0.21 (0.06–0.80) | 0.15 (0.04–0.55) | 0.36 (0.11–1.22) | 0.17 (0.07–0.43) | 1.00 (self-comparison) | 0.29 (0.06–1.51) | 0.25 (0.05–1.23) | 0.08 (0.02–0.35) | 0.13 (0.03–0.65) | 0.11 (0.02–0.62) | 0.13 (0.03–0.62) |
| 9. RIF1200 | 2.51 (0.80–7.87) | 1.38 (0.40–4.77) | 0.82 (0.24–2.80) | 0.73 (0.20–2.65) | 0.51 (0.14–1.83) | 1.23 (0.37–4.02) | 0.57 (0.14–2.40) | 3.40 (0.66–17.45) | 1.00 (self-comparison) | 0.83 (0.37–1.88) | 0.26 (0.06–1.17) | 0.43 (0.09–2.16) | 0.39 (0.07–2.07) | 0.44 (0.09–2.05) |
| 10. RIF1800 | 3.02 (0.99–9.18) | 1.66 (0.49–5.58) | 0.99 (0.30–3.27) | 0.87 (0.25–3.10) | 0.61 (0.17–2.14) | 1.47 (0.46–4.70) | 0.69 (0.17–2.81) | 4.08 (0.81–20.53) | 1.20 (0.53–2.71) | 1.00 (self-comparison) | 0.31 (0.07–1.38) | 0.52 (0.11–2.54) | 0.47 (0.09–2.43) | 0.52 (0.11–2.41) |
| 11. TR-RIFLZD | 9.84 (3.59–26.93) | 5.40 (1.77–16.53) | 3.21 (1.07–9.68) | 2.85 (0.88–9.21) | 1.98 (0.62–6.36) | 4.80 (1.66–13.85) | 2.24 (0.60–8.45) | 13.31 (2.84–62.35) | 3.91 (0.85–17.94) | 3.26 (0.73–14.63) | 1.00 (self-comparison) | 1.70 (0.89–3.22) | 1.52 (0.69–3.35) | 1.71 (1.08–2.72) |
| 12. TR-RIFCLO | 5.80 (1.88–17.94) | 3.19 (0.93–10.89) | 1.90 (0.56–6.39) | 1.68 (0.47–6.04) | 1.17 (0.33–4.17) | 2.83 (0.87–9.17) | 1.32 (0.32–5.48) | 7.85 (1.54–39.91) | 2.31 (0.46–11.49) | 1.92 (0.39–9.39) | 0.59 (0.31–1.12) | 1.00 (self-comparison) | 0.90 (0.35–2.30) | 1.01 (0.51–2.01) |
| 13. TR-RPTLZDLVX | 6.46 (1.91–21.89) | 3.55 (0.96–13.20) | 2.11 (0.58–7.75) | 1.87 (0.48–7.29) | 1.30 (0.34–5.04) | 3.15 (0.89–11.16) | 1.47 (0.33–6.57) | 8.75 (1.61–47.43) | 2.57 (0.48–13.67) | 2.14 (0.41–11.18) | 0.66 (0.30–1.45) | 1.11 (0.44–2.85) | 1.00 (self-comparison) | 1.12 (0.49–2.58) |
| 14. TR-BDQLZD | 5.75 (2.03–16.24) | 3.16 (1.00–9.93) | 1.88 (0.61–5.82) | 1.66 (0.50–5.53) | 1.16 (0.35–3.82) | 2.80 (0.94–8.33) | 1.31 (0.34–5.05) | 7.78 (1.63–37.18) | 2.29 (0.49–10.70) | 1.91 (0.42–8.73) | 0.58 (0.37–0.93) | 0.99 (0.50–1.97) | 0.89 (0.39–2.04) | 1.00 (self-comparison) |

Node key: 1. STD = 6-month standard; 2. S31-RPTMOX = 4-month RPT+MOX (Study 31/A5349; daily); 3. S31-RPT = 4-month RPT (Study 31/A5349; daily); 4. REMOX-H = 4-month MOX (H-subst; REMoxTB); 5. REMOX-E = 4-month MOX (E-subst; REMoxTB); 6. GAT = 4-month GAT (OFLOTUB); 7. RIFA-4 = 4-month RPT+MOX (RIFAQUIN; twice-weekly continuation); 8. RIFA-6 = 6-month RPT+MOX (RIFAQUIN; once-weekly continuation); 9. RIF1200 = 4-month RIF 1,200 mg (RIFASHORT); 10. RIF1800 = 4-month RIF 1,800 mg (RIFASHORT); 11. TR-RIFLZD = intended 8-week high-dose RIF+LZD; 12. TR-RIFCLO = intended 8-week high-dose RIF+CLO; 13. TR-RPTLZDLVX = intended 8-week RPT+LZD+LVX; 14. TR-BDQLZD = intended 8-week BDQ+LZD.

Model: frequentist fixed-effect consistency NMA on the log-risk-ratio scale with multi-arm covariance retained. The 14-node network had no residual degrees of freedom for a common heterogeneity variance. No continuity correction was required for relapse/recurrence-related outcomes.

**Panel D. League table for All-cause mortality**

| **Row treatment** | **1. STD** | **2. S31-RPTMOX** | **3. S31-RPT** | **4. REMOX-H** | **5. REMOX-E** | **6. GAT** | **7. RIFA-4** | **8. RIFA-6** | **9. RIF1200** | **10. RIF1800** | **11. TR-RIFLZD** | **12. TR-RIFCLO** | **13. TR-RPTLZDLVX** | **14. TR-BDQLZD** |
| --- | --- | --- | --- | --- | --- | --- | --- | --- | --- | --- | --- | --- | --- | --- |
| 1. STD | 1.00 (self-comparison) | 0.95 (0.43–2.06) | 1.10 (0.49–2.49) | 1.09 (0.55–2.19) | 1.33 (0.63–2.78) | 1.89 (0.64–5.60) | 0.50 (0.19–1.32) | 0.86 (0.29–2.53) | 0.62 (0.21–1.87) | 1.67 (0.40–6.92) | 1.02 (0.23–4.41) | 3.04 (0.16–58.14) | 0.55 (0.08–3.63) | 7.31 (0.38–140.50) |
| 2. S31-RPTMOX | 1.06 (0.48–2.30) | 1.00 (self-comparison) | 1.17 (0.53–2.59) | 1.16 (0.41–3.28) | 1.40 (0.48–4.11) | 1.99 (0.52–7.60) | 0.53 (0.15–1.83) | 0.91 (0.24–3.43) | 0.66 (0.17–2.53) | 1.77 (0.35–8.93) | 1.07 (0.20–5.65) | 3.21 (0.15–67.95) | 0.58 (0.08–4.47) | 7.72 (0.36–164.18) |
| 3. S31-RPT | 0.91 (0.40–2.04) | 0.86 (0.39–1.90) | 1.00 (self-comparison) | 0.99 (0.34–2.89) | 1.20 (0.40–3.61) | 1.71 (0.44–6.64) | 0.45 (0.13–1.61) | 0.78 (0.20–3.00) | 0.56 (0.14–2.22) | 1.52 (0.30–7.78) | 0.92 (0.17–4.93) | 2.75 (0.13–58.76) | 0.50 (0.06–3.88) | 6.62 (0.31–141.99) |
| 4. REMOX-H | 0.91 (0.46–1.83) | 0.87 (0.30–2.46) | 1.01 (0.35–2.94) | 1.00 (self-comparison) | 1.21 (0.57–2.57) | 1.73 (0.47–6.28) | 0.46 (0.14–1.51) | 0.79 (0.22–2.84) | 0.57 (0.15–2.09) | 1.53 (0.32–7.44) | 0.93 (0.18–4.72) | 2.78 (0.13–57.65) | 0.50 (0.07–3.76) | 6.68 (0.32–139.31) |
| 5. REMOX-E | 0.75 (0.36–1.58) | 0.71 (0.24–2.09) | 0.83 (0.28–2.50) | 0.82 (0.39–1.75) | 1.00 (self-comparison) | 1.42 (0.38–5.30) | 0.38 (0.11–1.28) | 0.65 (0.18–2.40) | 0.47 (0.12–1.77) | 1.26 (0.25–6.25) | 0.77 (0.15–3.96) | 2.29 (0.11–48.00) | 0.42 (0.05–3.14) | 5.51 (0.26–115.99) |
| 6. GAT | 0.53 (0.18–1.57) | 0.50 (0.13–1.91) | 0.59 (0.15–2.28) | 0.58 (0.16–2.11) | 0.70 (0.19–2.62) | 1.00 (self-comparison) | 0.27 (0.06–1.14) | 0.46 (0.10–2.11) | 0.33 (0.07–1.55) | 0.89 (0.15–5.31) | 0.54 (0.09–3.35) | 1.61 (0.07–37.41) | 0.29 (0.03–2.57) | 3.87 (0.17–90.38) |
| 7. RIFA-4 | 1.99 (0.76–5.23) | 1.89 (0.55–6.52) | 2.20 (0.62–7.77) | 2.18 (0.66–7.16) | 2.64 (0.78–8.93) | 3.76 (0.88–16.11) | 1.00 (self-comparison) | 1.71 (0.69–4.29) | 1.24 (0.29–5.37) | 3.34 (0.60–18.57) | 2.03 (0.35–11.73) | 6.05 (0.27–135.14) | 1.10 (0.13–9.12) | 14.56 (0.65–326.50) |
| 8. RIFA-6 | 1.16 (0.40–3.41) | 1.10 (0.29–4.16) | 1.28 (0.33–4.95) | 1.27 (0.35–4.58) | 1.54 (0.42–5.70) | 2.19 (0.47–10.14) | 0.58 (0.23–1.46) | 1.00 (self-comparison) | 0.72 (0.15–3.38) | 1.95 (0.33–11.56) | 1.18 (0.19–7.30) | 3.53 (0.15–81.77) | 0.64 (0.07–5.61) | 8.49 (0.37–197.55) |
| 9. RIF1200 | 1.61 (0.53–4.84) | 1.52 (0.39–5.86) | 1.77 (0.45–6.98) | 1.76 (0.48–6.47) | 2.13 (0.57–8.04) | 3.03 (0.64–14.27) | 0.81 (0.19–3.49) | 1.38 (0.30–6.46) | 1.00 (self-comparison) | 2.69 (0.72–10.01) | 1.63 (0.26–10.24) | 4.88 (0.21–114.01) | 0.89 (0.10–7.86) | 11.74 (0.50–275.43) |
| 10. RIF1800 | 0.60 (0.14–2.47) | 0.57 (0.11–2.85) | 0.66 (0.13–3.38) | 0.65 (0.13–3.17) | 0.79 (0.16–3.93) | 1.13 (0.19–6.74) | 0.30 (0.05–1.67) | 0.51 (0.09–3.05) | 0.37 (0.10–1.38) | 1.00 (self-comparison) | 0.61 (0.08–4.68) | 1.81 (0.07–47.99) | 0.33 (0.03–3.48) | 4.37 (0.16–115.93) |
| 11. TR-RIFLZD | 0.98 (0.23–4.27) | 0.93 (0.18–4.90) | 1.09 (0.20–5.81) | 1.08 (0.21–5.46) | 1.31 (0.25–6.75) | 1.86 (0.30–11.54) | 0.49 (0.09–2.86) | 0.85 (0.14–5.23) | 0.61 (0.10–3.84) | 1.65 (0.21–12.69) | 1.00 (self-comparison) | 2.99 (0.16–57.20) | 0.54 (0.08–3.57) | 7.19 (0.37–138.23) |
| 12. TR-RIFCLO | 0.33 (0.02–6.30) | 0.31 (0.01–6.59) | 0.36 (0.02–7.76) | 0.36 (0.02–7.47) | 0.44 (0.02–9.16) | 0.62 (0.03–14.43) | 0.17 (0.01–3.69) | 0.28 (0.01–6.55) | 0.20 (0.01–4.78) | 0.55 (0.02–14.57) | 0.33 (0.02–6.40) | 1.00 (self-comparison) | 0.18 (0.01–4.36) | 2.41 (0.05–120.16) |
| 13. TR-RPTLZDLVX | 1.81 (0.28–11.93) | 1.72 (0.22–13.18) | 2.00 (0.26–15.58) | 1.98 (0.27–14.77) | 2.41 (0.32–18.22) | 3.42 (0.39–30.14) | 0.91 (0.11–7.56) | 1.56 (0.18–13.67) | 1.13 (0.13–10.01) | 3.04 (0.29–32.12) | 1.84 (0.28–12.13) | 5.51 (0.23–132.41) | 1.00 (self-comparison) | 13.26 (0.55–319.88) |
| 14. TR-BDQLZD | 0.14 (0.01–2.63) | 0.13 (0.01–2.75) | 0.15 (0.01–3.24) | 0.15 (0.01–3.12) | 0.18 (0.01–3.83) | 0.26 (0.01–6.03) | 0.07 (0.00–1.54) | 0.12 (0.01–2.74) | 0.09 (0.00–2.00) | 0.23 (0.01–6.08) | 0.14 (0.01–2.67) | 0.42 (0.01–20.77) | 0.08 (0.00–1.82) | 1.00 (self-comparison) |

Node key: 1. STD = 6-month standard; 2. S31-RPTMOX = 4-month RPT+MOX (Study 31/A5349; daily); 3. S31-RPT = 4-month RPT (Study 31/A5349; daily); 4. REMOX-H = 4-month MOX (H-subst; REMoxTB); 5. REMOX-E = 4-month MOX (E-subst; REMoxTB); 6. GAT = 4-month GAT (OFLOTUB); 7. RIFA-4 = 4-month RPT+MOX (RIFAQUIN; twice-weekly continuation); 8. RIFA-6 = 6-month RPT+MOX (RIFAQUIN; once-weekly continuation); 9. RIF1200 = 4-month RIF 1,200 mg (RIFASHORT); 10. RIF1800 = 4-month RIF 1,800 mg (RIFASHORT); 11. TR-RIFLZD = intended 8-week high-dose RIF+LZD; 12. TR-RIFCLO = intended 8-week high-dose RIF+CLO; 13. TR-RPTLZDLVX = intended 8-week RPT+LZD+LVX; 14. TR-BDQLZD = intended 8-week BDQ+LZD.

Model: frequentist fixed-effect consistency NMA on the log-risk-ratio scale with multi-arm covariance retained. Because two TRUNCATE-TB arms reported zero deaths, a 0.5 continuity correction was applied to all TRUNCATE-TB arms and their common standard comparator for the mortality model only. Estimates and P-scores are exploratory because events were sparse.

League-table note: Diagonal cells represent self-comparisons, for which RR = 1.00 by definition; these cells are not model-estimated effects, bibliographic references, or missing data.

Abbreviations: CI, confidence interval; E-subst, ethambutol substitution; GAT, gatifloxacin; H-subst, isoniazid substitution; MOX, moxifloxacin; NMA, network meta-analysis; RIF, rifampicin; RPT, rifapentine; RR, risk ratio.

**Table S4. Estimability of inconsistency and heterogeneity assessments**

| **Assessment** | **Requirement** | **Observed network** | **Conclusion** |
| --- | --- | --- | --- |
| Design-by-treatment interaction | Residual df from independent designs | 14 nodes; 13 independent basic contrasts; 0 df | Not estimable |
| Node splitting | Independent direct and indirect evidence | No independent closed loop | Not estimable |
| Network heterogeneity | Residual df for common τ² | 0 df | Not estimable; fixed-effect model |
| Pairwise heterogeneity | Multiple shortened comparisons | 12 comparisons; 6 trials; I²=39.9%; Q-test P=0.08 | Moderate heterogeneity |

Structural triangles generated by multi-arm trials were consistent by construction and did not supply independent evidence for incoherence assessment. The absence of estimable incoherence should not be interpreted as evidence that all assumptions were verified. The 14-node network contained 13 independent basic contrasts, leaving zero residual degrees of freedom for estimating a common network heterogeneity variance.

**Figure S2. Sensitivity analyses for the pairwise meta-analysis of trial-defined unfavorable long-term outcomes.**

The figure compares the primary DerSimonian-Laird random-effects estimate with the fixed-effect estimate, the Hartung-Knapp-Sidik-Jonkman sensitivity estimate, and the random-effects estimate after exclusion of TRUNCATE-TB. All analyses compare treatment-shortening regimens with the daily 6-month standard regimen. RR > 1 indicates a higher risk of a trial-defined unfavorable long-term outcome with treatment shortening. Shared comparator groups were divided equally across eligible comparisons for weighting while preserving the original control risks. The 6-month RIFAQUIN intervention remains excluded from the shortened-duration pool and is retained in the NMA.

**Figure S3. Descriptive P-scores for treatment success.**

Higher values indicate a more favorable ranking. Rankings are descriptive and must be interpreted with the RRs and 95% CIs in Table S3. TRUNCATE-TB estimates refer to conventional regimen efficacy.

**Figure S4. Descriptive P-scores for relapse/recurrence-related outcomes.**

Higher values indicate a more favorable ranking. Rankings are descriptive and must be interpreted with the RRs and 95% CIs in Table S3. Relapse was the main driver of unfavorable outcomes in TRUNCATE-TB.

**Figure S5. Descriptive P-scores for all-cause mortality.**

Higher values indicate a more favorable ranking. Mortality rankings are unstable and not clinically interpretable because events were sparse and continuity correction was required for TRUNCATE-TB.

**Figure S6. Risk-of-bias assessment for the primary long-term efficacy result used from each included randomized trial.**

Judgements are outcome-specific and refer to the primary long-term efficacy result used in this review. Study 31/A5349 and REMoxTB were rated overall low risk of bias; the other six trials had some concerns, and none was rated high risk. Open-label status alone was not used to assign some concerns. For Study 31/A5349, outcome measurement was rated low risk because microbiologists and the central trial-operations team determining outcome status were unaware of treatment assignment and trial week.

**Figure S7. Adapted CINeMA-style certainty assessment across outcomes.**

This adapted assessment did not use the CINeMA web application or a contribution matrix and should not be interpreted as a formal CINeMA assessment. Heterogeneity was not assessable in the 14-node fixed-effect network, and incoherence was not applicable because there was no independent closed loop. TRUNCATE-TB contributed additional indirectness and imprecision because it was a single adaptive trial with protocol-permitted extension and small or early-stopped experimental arms.
